# Multidimensional profiling of rugby league players: A systematic scoping review and expert Delphi consensus

**DOI:** 10.1371/journal.pone.0327867

**Published:** 2025-08-20

**Authors:** Sam Wild, Cameron Owen, Ben Jones, Sam McCormack, Omar Heyward, Sean Scantlebury, Dave Rotheram, Neil McCarthy, Kevin Till

**Affiliations:** 1 Carnegie Applied Rugby Research (CARR) Centre, Carnegie School of Sport, Leeds Beckett University, Leeds, United Kingdom; 2 England Performance Unit, Rugby Football League, Manchester, United Kingdom; 3 Premiership Rugby, London, United Kingdom; 4 Division of Physiological Sciences and Health through Physical Activity, Lifestyle and Sport Research Centre, Department of Human Biology, Faculty of Health Sciences, University of Cape Town, Cape Town, South Africa; 5 School of Behavioural and Health Sciences, Faculty of Health Sciences, Australian Catholic University, Brisbane, Queensland, Australia; 6 Rugby Football Union, London, United Kingdom; 7 Leeds Rhinos Rugby League Club, Leeds, United Kingdom; Charles Sturt University, AUSTRALIA

## Abstract

Player profiling can aid talent identification and development by highlighting strengths and weaknesses, and evaluating training interventions. However, there is currently no consensus in rugby league on the qualities, skills, and characteristics (i.e., factors) which should be profiled, or the methods to use to assess these factors. Consequently, the aims of this two-part study were to 1) establish the most common factors and methods for profiling rugby league players, through a systematic scoping review, and 2) develop consensus on the factors and methods experts believe should be used when profiling rugby league players. In Part 1, a systematic scoping review of studies profiling rugby league players was conducted according to the PRISMA guideline for Scoping Reviews. In Part 2, a panel of 32 experts were invited to participate in a sequential three-round Delphi consensus, used to identify the factors that they believed should be profiled in rugby league players and associated methods of assessment. Part 1 identified 370 studies, which assessed varying numbers of factors from five higher order themes; physical (n = 247, 67%), health-related (n = 129, 35%), other (n = 60, 16%; e.g., playing experience, level of education), technical-tactical (n = 58, 16%), and psychological (n = 25, 7%). Only 3% of these studies featured female participants (n = 11). In Part 2, 120 factors were initially identified, of which 85 reached consensus (≥70% agreement). This included 22 physical, 22 psychological, 20 technical-tactical, 15 health-related, and six player information factors. Collectively, these findings evidence the multidimensional nature of talent in rugby league, highlighting a range of factors across several domains that should be considered when identifying and monitoring talent in the sport. Furthermore, technical-tactical and psychological factors were identified as areas for future research, due to the large number of factors which reached consensus in these areas and the comparatively low amount of research conducted in them.

## Introduction

Talent in sport is considered to be multidimensional and therefore constituted by a range of qualities, skills, and characteristics (henceforth referred to as ‘factors’) from different domains [1]. In rugby league, coaches have identified several physical, technical-tactical, and psychological factors which they believe are early indicators of high performance in youth players [2]. This reflects the multidimensional demands of the sport, which requires players to perform intermittent bouts of high intensity physical activity such as sprinting and jumping [3], alongside technical-tactical skills such as passing and tackling [4]. Consequently, physical [5–7], technical-tactical [8,9], and psychological factors [10] have all been shown to discriminate between playing standards in rugby league. This indicates that a range of factors influence how players are able to meet the demands of the game, highlighting the multidimensional nature of talent in rugby league.

Practitioners can utilise the systematic assessment of multidimensional factors to identify talent, profile athletes’ strengths and weaknesses and monitor the effectiveness of training interventions [11–13]. This can be achieved through a battery of objective or subjective assessments [14,15]. In rugby league, coaches have indicated that they believe player profiling that focuses solely on physical qualities is of limited utility as it does sufficiently encompass a player’s talent [16]. As such, recent calls have been made for profiling to be more multidimensional in nature [11,16]. To achieve this, an understanding of the factors to be assessed and the most appropriate methods for assessing these factors is necessary, however no studies to date have systematically mapped the factors and methods used to profile rugby league players, beyond their physical qualities [17]. Furthermore, applied practitioners often have contrasting perspectives to researchers in sport [18], therefore expert opinion can help create a broader understanding of multidimensional player profiling, by potentially identifying factors and methods beyond what has been used previously in research, whilst ensuring they are suitable for applied environments. Consequently, the aims of this two-part study were to 1) establish the most common factors and methods for profiling rugby league players, through a systematic scoping review, and 2) develop consensus on the factors and methods experts believe should be used to profile rugby league players. The achievement of these aims can guide the development of multidimensional player profiling in rugby league which can inform talent identification and development practices by better reflecting the multidimensional nature of talent in the sport. This can also guide future research direction by highlighting discrepancies between research focus and expert opinion when considering multidimensional talent in rugby league.

## Methods

This study employed a two-part design; Part 1 was a systematic scoping review of the research literature profiling rugby league players, whilst Part 2 was a sequential, three-round Delphi-based consensus process. Ethical approval for this study was granted by the Leeds Beckett University Local Research Ethics Committee, in line with the Research Ethics and Policy and Procedures of Leeds Beckett University (application reference: 111823).

### Part 1: Systematic scoping review

A systematic scoping review of studies that had profiled rugby league players was initially carried out in line with the Preferred Reporting Items for Systematic Reviews and Meta-Analyses (PRISMA) [19] (S1 File) and the PRISMA extension for scoping reviews guidelines [20] (S2 File). The protocol for this review was registered online on the Open Science Framework website (DOI 10.17605/OSF.IO/N29ER).

#### Literature search.

Literature searches were carried out in June 2022, on six electronic databases from their earliest records: CINAHL, MEDLINE, PsychInfo, PubMed, Scopus, SportDiscus. One search was conducted for each database. These databases were chosen based on recent reviews conducted in similar areas [21–23], with the aim of providing the broadest scope for searches.

Previous research suggested the higher order themes which rugby league players may be profiled in could include technical-tactical skills, physical qualities, psychological skills and characteristics, and physical health [2,24]. Consequently, consultation within the research team led to a list of search terms thought to comprehensively represent these higher order themes based on previous literature and the research team’s practical experience. The same search terms were applied to each database whereby the primary term ‘rugby league’ was combined with several secondary search terms representing higher order themes. The full search strategy used was “rugby league” AND (“speed” OR “power” OR “fitness” OR “strength” OR “physical” OR “anthro*” OR “endurance” OR “agility” OR “matur*” OR “accelerat*” OR “mental” OR “cognitive” OR “psychological” OR “hardiness” OR “motivat*” OR “mental toughness” OR “aggres*” OR “concentrate*” OR “attitude” OR “discipline” OR “techni*” OR “carry*” OR “pass*” OR “tackl*” OR “skill” OR “kick*” OR “collision*” OR “health*” OR “injur*” OR “illness*” OR “fatigue” OR “wellness” OR “well being” OR “well-being” OR “nutrition” OR “diet” OR “sleep” OR “social” OR “family” OR “peer” OR “media” OR “culture” OR “perform*” OR “ability*” OR “characteristic*” OR “profil*” OR “qualit*” OR “assess*” OR “test*” OR “evaluat*” OR “measur*”). This search strategy was applied to all fields within each database, with only English language studies from peer-reviewed journals included. Reference lists for each study were not screened.

#### Study selection.

The inclusion criteria were that studies had to measure the qualities (e.g., lower body strength), skills (e.g., tackling), characteristics (e.g., ethnicity), or status (e.g., injury status) of rugby league players and must be from peer-reviewed journals written in English. Studies investigating samples of athletes from sports other than rugby league, shortened formats of rugby league (e.g., 9-a-side rugby league; [25]), non-contact, and wheelchair rugby league were excluded. Grey literature and conference abstracts were also excluded. Studies which met all the inclusion criteria and none of the exclusion criteria were included in the review. The exclusion of studies focusing on wheelchair rugby league, shortened formats of the game, or non-contact rugby league was due to the potential for different factors being relevant to them compared to the 13-a-side game. There was no limitation on the age of players included in the systematic scoping review so that the broadest possible range of factors and methods could be identified, with subsequent analysis used to discern differences in research focus between youth and senior players.

#### Study screening.

The screening process was carried out by two members of the research team (SW and SM). The titles of search results from each database were collated in a Microsoft Excel (Microsoft Corporation, Washington, USA) spreadsheet. Duplicates were then removed using a bespoke script written in R Studio (V4.1.2, R Foundation for Statistical Computing, Vienna, Austria). Following the removal of duplicates, titles were screened independently by each researcher based on the inclusion and exclusion criteria. Any studies on which the researchers disagreed were discussed verbally to assess their appropriateness before making a final decision on their progress to the next stage of screening. This process was repeated for the abstracts of each study. The mean level of agreement between reviewers across the initial title and abstract review processes was 76%. Full text screening was carried out by one researcher (SW), as part of the data charting process.

The study screening and data charting process was initially carried out for 46 studies to assess its appropriateness. The initial search covered physical, technical-tactical, and psycho-social terms (Table 1) and data charting was carried out to the level of the specific factors assessed in each study. Following review by the research team, the decision was made to expand the search to include health and general terms (Table 1) to make the review broader in nature and reflect a more multidimensional approach. The data charting process was also carried out in greater detail to the level of the variables reported to provide a more comprehensive overview of what the studies assessed.

**Table 1:** Expert panel criteria.

Area of Work	Criteria	Time in Role
Rugby league coaching	Super League or NRL head coach	>5 years
	Super League or NRL head of youth/pathways	>5 years
	Senior or youth international head coach	>1 year
Sports Science/ Strength & Conditioning	Super League or NRL head of performance	>5 years
Medical	Super League or NRL head of medical/physiotherapy	>5 years
Psychology	Elite team sport practitioner	>5 years
Nutrition	Elite team sport practitioner	>5 years
Other sports	Elite team sports talent development role	>5 years
Research	Published >10 studies in a relevant area	N/A

NRL – National Rugby League competition.

#### Data charting.

A Microsoft Excel spreadsheet was used for extracting information from included studies. General study information was initially recorded, including year of publication, geographical location, and age, sex and playing standard of the sample used. The factors assessed in each study were recorded at multiple levels: the variables reported (e.g., one-repetition maximum), the specific factor (e.g., lower body strength), the general factor (e.g., strength), the higher order theme (e.g., physical), and the method used (e.g., back squat). For technical-tactical factors the setting in which the factor was assessed was also recorded (e.g., training drill, match).

Specific factors were classified based on the quality, skill, or characteristic that the variable reported was considered to represent (e.g., one-repetition maximum for a back squat was considered to represent lower body strength at a specific factor level). General factors were determined based on broader themes that the specific factors related to (e.g., lower body strength was related to strength at the general factor level). Higher order themes were selected based on broader research topics in rugby league, with factors classified based on the higher order theme they were deemed to represent most closely (e.g., lower body strength was considered to represent the ‘physical’ higher order theme). This process was carried out initially by the lead researcher (SW), before being reviewed by the research team (BJ, KT, SM).

#### Descriptive statistics.

All analysis was conducted using R Studio. The frequency of study characteristics (e.g., number of studies published in Australia) and the number of studies measuring different factors (e.g., number of studies measuring upper body strength) and using different methods (e.g., number of studies using bench press to measure upper body strength) were quantified to reflect the amount of research dedicated to specific areas.

### Part 2: Delphi consensus process

The second part of this study aimed to establish consensus on what experts recommended should be monitored as part of the multidimensional profile of a rugby league player and how these factors should be monitored. This was achieved through a sequential three-round online Delphi process; the first round focusing on idea generation followed by two rounds of voting to establish consensus [21,26].

#### Recruitment.

Participants were purposefully sampled based on their professional and academic experience [27]. Staff lists for professional rugby league clubs in Australia and England were systemically searched (where available) to identify potential participants. Table 1 outlines the criteria used to classify the experts who were contacted to participate in this study. These criteria were chosen to ensure a wide range of expertise and experience in rugby league, research, and other high-performance sports. Previous research has indicated that a diverse panel composition can lead to a wider range of opinions, meaning any consensus that is reached is likely to have greater validity [28]. Past studies have specified three to five years of professional experience to classify experts for a sports science-based Delphi panel, or more than three studies published in a relevant area for academics [24,29]. As such, inclusion criteria for experts working in applied roles was set to 5 years of relevant professional experience, aside from international head coaches as these roles represent the highest level of coaching in rugby league. Inclusion criteria for academics was set to 10 publications in a relevant topic area to ensure high-level experts were recruited. Participants were required to meet one or more of these criteria to be eligible to participate.

Participants were contacted via email or professional social media platforms (i.e., LinkedIn) to inform them of the study and invite them to participate, with the recruitment process beginning on Thursday 3^rd^ August 2023 and ending on Wednesday 23^rd^ August 2023. If participants stated an interest, a participant information sheet, two study infographics, and a participant consent form were sent. Those who agreed to participate provided written informed consent via questionnaires.

#### Panel composition.

Out of the 92 potential participants contacted to take part in the study, 32 (35%) consented to participate. Delphi panels typically contain 11–25 participants [30], making this a relatively large panel. This was due to the broad range of specialist areas participants were recruited from. Of the 32 who consented to participate, 26 (81%) completed all three rounds, four (13%) completed two rounds, and two participants (6%) completed one round. This exceeds the 75% retention rate evident in previous research [31].

Table 2 highlights the characteristics of the members of the expert panel. Participants represented seven broad professional areas, chosen to reflect the higher order themes identified in the systematic scoping review. Some participants were recruited from outside of rugby league to provide diverse perspectives on player profiling. The geographical areas represented by the panel reflect the countries in which rugby league is most popular, with 88% of participants working in either England or Australia. Four participants worked in both research-based and applied roles, four participants stated that they worked across more than one sport, and five participants did not state a sport that they worked in. The mean number of years participants had held their current or an equivalent role was 14.9 ± 7.0 years. Three members of the panel were female and 29 were male.

**Table 2:** Number of panel members (percentage of total) representing different professions, sports, and countries.

Participant Characteristics		Number of Participants (percentage of total)
*Professional Area*	Research	10 (31%)
	Youth development pathways	9 (28%)
	Physical performance	5 (16%)
	Medical	5 (16%)
	Sports coaching	3 (9%)
	Sports psychology	2 (6%)
	Sports nutrition	2 (6%)
*Sport*	Rugby league	17 (53%)
	Rugby union	4 (13%)
	Cricket	3 (9%)
	Soccer	2 (6%)
	Australian rules football	1 (3%)
	Olympic sports	1 (3%)
*Country of Work*	England	16 (50%)
	Australia	12 (38%)
	New Zealand	1 (3%)
	Wales	1 (3%)
	Ireland	1 (3%)
	Canada	1 (3%)

#### Delphi questionnaires.

All questionnaires were designed and distributed using Qualtrics (Qualtrics, Utah, USA). Participants were given seven days to complete each questionnaire, with a reminder sent to those who had not completed the questionnaire after four days. Results from each round were fed back to participants prior to the commencement of the following round [32]. Feedback documents were created using Microsoft Word (Microsoft Corporation, Washington, USA), featuring tables summarising the number and percentage of participants who scored a one, two, or three, with factors and methods that reached consensus highlighted in bold.

Participants were instructed to provide their responses on the basis that they were not limited by resources such as time, money, or equipment, and that their responses could be relevant to any context within rugby league (e.g., men’s or women’s rugby league, any time of season). The aim was to encourage a broad range of responses that would be relevant to cohorts across the whole sport.

#### Round 1.

Participants were initially sent a summary of the findings from the systematic scoping review which they were asked to read prior to beginning the Round 1 questionnaire (S3 File). This outlined the most common factors and methods identified within the literature. They were also sent a comprehensive list of the factors and methods that had been identified during the literature review, to provide some background in terms of the scope and nature of research profiling rugby league players. Findings from the systematic scoping review were shared with the Delphi panel prior to commencing the consensus process to account for some panellists’ potential lack of familiarity with existing research knowledge, given that 69% of the panel worked in applied, rather than research-based roles. It was also thought that sharing the results from the systematic scoping review would provide some context around the broad, multidimensional scope of the project to encourage a wide range of responses.

The Round 1 questionnaire asked participants to list the factors which they believe should be monitored as part of a multi-dimensional profile of a rugby league player. Participants were also asked to provide a brief definition of the factors outlined, optionally provide a rationale for their choice, and suggest methods for measuring each factor.

Each response for the first questionnaire was manually assessed, with factors, methods, and associated definitions extracted using a Microsoft Excel spreadsheet. Following the extraction of this data, responses were analysed for consistent themes based on repetition of factors and methods or definitions which identified overlapping concepts. Subsequently, similar factors and methods were combined, with the most specific level of a given factor (e.g., ‘lower body muscular power’ versus ‘muscular power’) retained. Definitions were also summarised to provide one concise definition that accounted for the range of responses for each factor. In the case that no definition was provided for a factor, the lead researcher (SW) provided one. The factors, definitions, and methods identified through this process were taken forward into the Round 2 questionnaire.

Responses from Round 1 were categorised into five higher order themes by the lead researcher (SW), chosen to reflect the higher order themes identified in the literature review; physical, psychological, technical-tactical, health-related, and player information. Factors were classified as physical if they reflected physical performance qualities (e.g., lower body strength), psychological if they related a player’s state of mind or psychological skills and characteristics (e.g., self-awareness), health-related if they related to a player’s health status, wellbeing, or factors that could directly contribute to these (e.g., sleep quality), technical-tactical if they related to rugby-specific skills or abilities (e.g., ball carrying ability), and player information if they reflected specific characteristics of the player or their life (e.g., birth quartile).

#### Round 2.

The Round 2 questionnaire first asked participants to rate their level of agreement regarding whether individual factors should be included in the profile of a rugby league player. This was done using a three-point Likert scale whereby 1 represented *disagree*, 2 represented *neither agree nor disagree* and 3 represented *agree*. A three-point scale was chosen as they are perceived as quicker and easier when using a large number of items [33]. Participants were also given an ‘outside my area of expertise’ option to abstain from voting; this was due to the diversity of the panel’s professional backgrounds. Participants were also able to leave general comments at the end of the questionnaire. The same scoring system was also used to rate level of agreement regarding whether listed methods should be used to monitor the various factors. Previous work has shown that Delphi consensus thresholds range from 50–97% agreement, with a median score of 75% [30]. Consequently, the level of consensus for factors and methods was set at ≥70% agreement to reflect the diverse nature of the panel which is likely to encourage more diverse opinions, thus reducing the level of agreement, whilst also reflecting similar studies conducted in the same area [21,24,34]. Anything which did not reach consensus was carried forward into Round 3.

#### Round 3.

The Round 3 questionnaire and summary feedback utilised the same format as Round 2. Only factors and methods which did not reach consensus in Round 2 were carried forward into Round 3. New methods were also included in the Round 3 questionnaire, following comments made in the final section of the Round 2 questionnaire. This was done to ensure the widest possible range of methods was available to vote on, based on participants’ expert opinion, therefore making the scope of the findings from the study as broad as possible. Participants were sent a comprehensive list of factors and associated methods which reached consensus over the three rounds following Round 3.

## Results

### Part 1: Systematic scoping review

**Fig 1:**
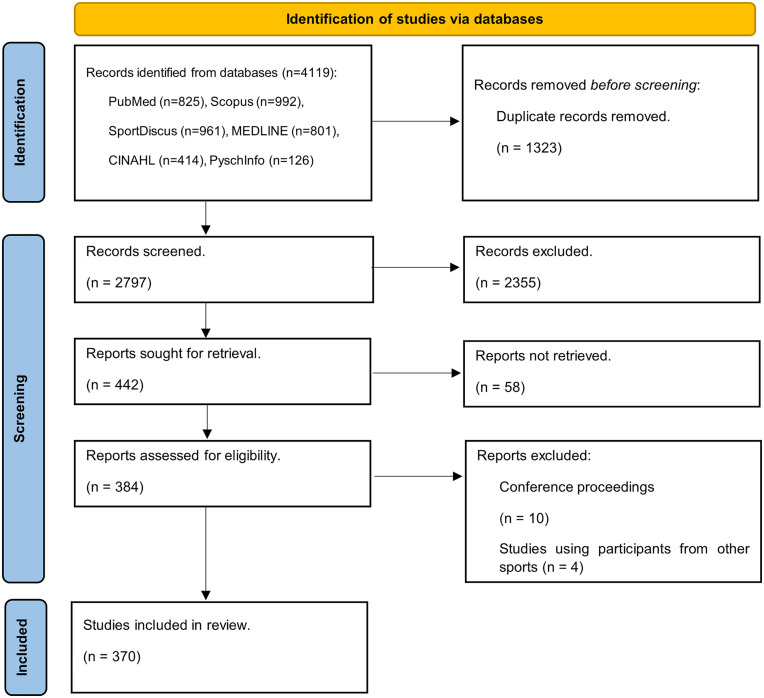
Flow chart of articles from identification to inclusion. Taken from Page, McKenzie [19].

The literature search initially identified 4,119 records, 1,323 of which were duplicates, leaving 2,797 unique records. Following title, abstract and full text screening there were 370 studies which met the eligibility criteria and had full text available (Fig 1).

#### Demographic information.

From the 370 studies included within the systematic scoping review, most studies assessed adult senior players (n = 275, 74%), whilst 35% of studies (n = 133) featured youth players across a range of age groups from U6 to U20s. Youth players were defined as any player who played within an age-group based structure (e.g., U18s), whilst senior players were classified as those whose age group was not specified. In addition, six studies focused on retired players (2%). Most studies had male participants (n = 363, 98%), whilst only 11 (3%) studies featured female participants.

#### Higher order themes.

Table 3 presents the prevalence of the five higher order themes identified in the literature, alongside their subsidiary specific factors, based on the age category of the sample in each study. Studies measuring physical factors were the most common overall (n = 247, 67%), and in both senior (n = 171, 62%) and youth cohorts (n = 96, 72%), however they formed a greater proportion of studies assessing youth players. Physical factors typically related to players’ physical performance qualities. Health factors were the second most common overall (n = 129, 35%) and were classified based on whether they related to a players’ physical health, wellbeing, or medical conditions. Of the studies assessing health-related factors, 78% featured senior players whilst only 30% assessed youth players. Fifty-eight studies overall assessed factors classified under the technical-tactical theme (16%), which typically consisted of assessments of players’ rugby-specific skills (e.g., tackling, ball carrying, passing). Fifty-two of these studies assessed senior players (90%), compared to 13 assessing youth players (22%). The least prevalent higher order theme overall (n = 25), in senior (n = 18), and in youth cohorts (n = 3) was psychological, which featured in just 7% of total studies. Psychological factors related to a players’ mental state or mental performance. Some factors were deemed to fall outside of the primary higher order themes but did not occur frequently enough to constitute their own higher order theme, in which case they were categorised as ‘other’ (n = 60, 16%). These factors largely related to information about the player or their background, such as their playing history or their social background.

**Table 3:** The number of studies measuring different higher order themes and associated specific factors split based on age category.

Higher order theme (overall number of studies, percentage of total)	Number of studies in senior players (percentage of total for this cohort)	Number of studies in youth players (percentage of total for this cohort)	Specific Factor (overall number of studies, percentage of total higher order theme)	Number of studies in senior players (percentage of total for this cohort and higher order theme)	Number of studies in youth players (percentage of total for this cohort and higher order theme)
Physical (n = 247, 67%)	n = 171 (62%)	n = 96 (72%)	Anthropometry (n = 133, 54%)	88 (51%) [5,6,35–120]	56 (58%) [5,6,8,14,41,58–60,75,81,83,87,90,94,96,97,116,119,121–158]
			Muscular power (n = 129, 52%)	82 (48%) [5–7,36,46–51,53–56,59–62,66,69,70,72–75,77,80,81,87,89–94,98–108,112,114,159–192]	61 (64%) [5–8,14,59,60,75,81,87,90,94,121,123,125–129,131,132,134–153,155–158,171,178,179,189,190,193–203]
			Sprinting (n = 90, 36%)	46 (27%) [36,39,43,46,48,50,51,54,55,57,60,66,70–72,74,87–94,97,99,00,103–107,111–114,119,165,181,183,184,188,191,204–206]	48 (50%) [8,14,50,60,87,90,94,97,119,121,123,125,126,128,129,131,132,134–153, 155–157,195–197,199–202,207]
			Cardiovascular fitness (n = 87, 35%)	47 (27%) [36,43,46,48,50,51,60,70–74,79,80,87–92,94,99–101,104–107,114,117–119,175,182,183,208–219]	46 (48%) [14,60,87,90,94,119,121,123,126–129,132,134–139,141–148,150–153,155–157,195,197,198,200,218,220–226]
			Muscular strength (n = 83, 34%)	61 (36%) [5,6,36,39,45,47,48,52–54,57,59,61–63,67,68,70,71,73,75,81,83,88,98,100–103,111–114,120,159,161,162,165,167,168,170,171,177–179,181,182,184–186,188–192,204,212,227–230]	33 (34%) [5,6,14,59,75,81,83,121,125,129–131,139,141,153,156,158,171,178,179,189,190,196,199,202,203,223,228,231–235]
			Agility (n = 53, 21%)	28 (16%) [46,50,51,54,55,60,66,72,74,88–90,92–94,99,104–106,112,119,165,205,206,236–239]	31 (32%) [8,60,90,94,119,123,127,128,132,134–138,140,142,144–147,150,151,155,157,195,197,198,200,236,237,240]
			Physical fatigue (n = 36, 15%)	27 (16%) [44,48,80,117,118,209,211,241–260]	9 (9%) [197,223–225,261–265]
			Running momentum (n = 12, 5%)	5 (3%) [73,165,188,218,266]	8 (8%) [129,138,139,141,153,156,200,218]
			Muscular strength endurance (n = 12, 5%)	8 (5%) [61,70,71,88,101,182,212,267]	4 (4%) [131,144,152,198]
			Hormonal status (n = 10, 3%)	10 (6%) [48,63,80,241,242,249,252,255,268,269]	0 (0%)
			Movement competency (n = 5, 2%)	2 (1%) [6,90]	5 (5%) [6,90,133,140,199]
			Muscular power endurance (n = 2, 1%)	1 (<1%) [88]	1 (1%) [144]
			Balance (n = 1, < 1%)	1 (<1%) [108]	0 (0%)
Health (n = 129, 35%)	n = 100 (36%)	n = 39 (29%)	Injury (n = 90, 70%)	74 (74%) [35,45,46,65,68,70,78,82,87,96,97,105,107–109,183,214,239,270–325]	25 (64%) [87,96,97,133,135,146,154,272,282,285,287,289–291,294,302,310,314,325–331]
			Sleep (n = 14, 11%)	11 (11%) [76,85,248,250,332–338]	1 (3%) [337]
			Diet (n = 14, 11%)	7 (7%) [38,42,63,84,108,209,339]	5 (13%) [124,328,340–342]
			Athlete wellness (n = 14, 11%)	6 (6%) [254–257,323,343]	8 (21%) [196,197,224,225,262–265]
			Illness (n = 4, 3%)	3 (3%) [250,343,344]	1 (3%) [345]
			Fatigue (n = 3, 2%)	3 (3%) [79,248,271]	0 (0%)
			Hydration (n = 2, 2%)	2 (2%) [40,114]	0 (0%)
			Cardiovascular health (n = 1, 1%)	1 (1%) [85]	0 (0%)
Other (n = 60, 16%)	n = 40 (15%)	n = 19 (14%)	Playing experience (n = 42, 70%)	32 (80%) [38,50,65,68,70,74,76,82,84,91–94,97,101,104,106–109,183,186,237,272,276,301,307,317,346–349]	10 (53%) [8,94,97,127,145,147,198,237,272,328]
			Age (n = 8, 13%)	4 (10%) [52,103,276,350]	4 (21%) [123,137,151,351]
			Social background (n = 8, 13%)	6 (15%) [97,301,303,350,352,353]	3 (16%) [97,328,354]
			Education n = 4, 7%)	3 (8%) [84,273,301]	0 (0%)
			Equipment use (n = 4, 7%)	3 (8%) [97,303,307]	2 (11%) [97,328]
			Lived experiences (n = 2, 3%)	2 (5%) [355,356]	1 (5%) [355]
			Preferred handedness (n = 2, 3%)	1 (3%) [301]	0 (0%)
			Training history (n = 2, 3%)	0 (0%)	2 (11%) [139,198]
			Employment history (n = 1, 2%)	1 (3%) [301]	0 (0%)
			Nationality (n = 1, 2%)	0 (0%)	1 (5%) [351]
			Primary language (n = 1, 2%)	1 (3%) [301]	0 (0%)
			Role models (n = 1, 2%)	0 (0%)	1 (5%) [357]
Technical-Tactical (n = 58, 16%)	n = 52 (19%)	n = 13 (10%)	Defensive involvements (n = 26, 45%)	24 (46%) [73,98,102,104,186,192,248,256,278,305,309,317,358–369]	4 (31%) [148,262,359,368]
			Offensive involvements (n = 26, 45%)	25 (48%) [73,104,186,244,248,256,288,309,352,358–373]	5 (38%) [148,359,368,371,373]
			Defensive skills (n = 24, 41%)	21 (40%) [9,47,50,55,71,72,81,86,93,98,99,102,104,106,162,170,192,239,317,374,375]	5 (38%) [8,9,81,376,377]
			Offensive skills (n = 11, 19%)	9 (17%) [9,50,99,104,106,239,378–380]	3 (23%) [9,377,381]
			Discipline (n = 6, 10%)	6 (12%) [73,359,361,363,366,369]	1 (8%) [359]
			General skills (n = 2, 3%)	2 (4%) [4,99]	1 (8%) [4]
Psychological (n = 25, 7%)	n = 18 (7%)	n = 3 (2%)	Mental health (n = 13, 52%)	8 (44%) [79,248,271,316,348,349,382,383]	2 (67%) [354,384]
			Psychological skills & characteristics (n = 8, 32%)	3 (17%) [380,385,386]	2 (67%) [152,354]
			Brain function (n = 5, 20%)	5 (28%) [104,106,239,370,380]	0 (0%)
			Mental fatigue (n = 2, 8%)	2 (11%) [245,246]	0 (0%)
			Personality traits (n = 1, 4%)	0 (0%)	1 (33%) [354]

Table 4 shows the number of studies measuring factors from multiple higher order themes, based on the age category of the sample. In total, 256 studies only assessed factors from one higher order theme (69%), of which 156 measured only physical, 61 measured only health factors, 25 measured only technical-tactical factors, eight studies measured only factors classified as ‘other’, and six studies only measured psychological factors. In contrast to this, only 30 studies were identified that assessed factors from three or more higher order themes (8%). When comparing between senior and youth cohorts, the number of studies assessing two higher order themes was 87% higher in senior samples, whilst the number of studies assessing three or more higher order themes was more than five times greater in senior players. Furthermore, no studies featuring youth players were identified which assessed four or more higher order themes. In studies assessing three higher themes (n = 25, 7%), the most common combination was physical, health, and ‘other’ (n = 13, 4%). In studies assessing four higher order themes (n = 5, 1%), two studies assessed physical, health, technical-tactical, and psychological factors, two studies assessed physical, psychological, technical-tactical, and ‘other’ factors and one study assessed physical, health, psychological, and ‘other’ factors.

**Table 4:** The number of studies measuring different numbers of higher order themes based on age category.

Number of Higher Order Themes (overall number of studies, percentage of total)	Number of studies in senior players (percentage of total for this cohort)	Number of studies in youth players (percentage of total for this cohort)
1 (n = 256, 69%)	192 (70%) [4–7,9,36,37,39,41,43,44,48,49,51,53,54,56–62,64,66,67,69,75,77,80,83,88–90,95,100,110–113,115–120,159–161,163–169,171–182,184,185,187–191,204–206,208,210–213,215–219,227–230,236,238,241–243,247,249,251–253,258–260,266–270,274,275,277,279–287,289–300,302,304,306,309–315,318–325,332–339,343,344,346,347,350,353,355,358–369,371–375,378,379,382,383,385–388]	99 (74%) [4–7,9,14,41,58–60,75,83,90,116,119,121,122,125,126,128–132,134,136,138,140–144,149,150,153,155–158,171,178,179,189,190,193–195,199–203,207,218,220–223,226,228,231–236,240,261,282,285,287,289–291,294,302,310,314,325,327,329–331,337,340–342,345,351,355,357,359,368,371,373,376,377,381,384]
2 (n = 84, 23%)	58 (21%) [35,40,42,45,47,52,55,63,71–74,78,81,85–87,91,92,94,96,98,99,101–103,105,114,127,162,170,192,209,214,237,244–246,250,254,255,257,271–273,276,278,288,301,303,305,307,309,316,348,349,352,380]	31 (24%) [81,87,94,96,123,124,127,133,135,137,139,145–148,151,152,154,196–198,224,225,237,263–265,272,326,328,354]
3 (n = 25, 7%)	19 (7%) [38,50,65,68,70,76,79,82,84,93,97,107–109,183,186,256,317,370]	3 (2%) [8,97,262]
4 (n = 5, 1%)	4 (2%) [104,106,239,248]	0 (0%)

#### Physical factors.

The most prevalent physical factors were anthropometric measures (n = 133, 54%). Most studies featuring anthropometric factors measured players’ body mass (n = 126, 95%), primarily using digital scales (n = 79, 63%). Ninety-nine studies also measured players’ height (74%), almost exclusively via a stadiometer (n = 72, 73%). It should be noted that for both body mass and height, several studies did not specify the methods used. Seventy-nine studies also measured players’ body composition (59%), utilizing a range of measures including skinfold calipers (n = 53, 67%), Dual Energy X-Ray Absorptiometry scans (n = 11, 14%), and bio-electrical impedance scales (n = 6, 8%).

Muscular power was another common physical factor (n = 129, 52%). Most of these studies measured lower body muscular power (n = 114, 88%), typically via assessments of different jump variations such as the countermovement jump (n = 60, 53%), vertical jump (n = 34, 30%) and jump squat (n = 19, 17%). Jump variations were measured using a range of equipment such as force plates (n = 31, 27%), yardsticks (n = 28, 25%), and jump mats (n = 22, 19%). Assessments of upper body muscular power were less common (n = 47, 36%) and were often assessed via med ball throws (n = 19, 38%) and bench throws (n = 16, 34%).

Sprinting was assessed in 90 studies (36%), most of which measured maximal speed capabilities (n = 87, 97%), although three analysed sprint mechanics (3%). Speed was measured over a range of distances including 10m (n = 4, 4%), 20m (n = 26, 29%), 30m (n = 7, 8%), 40m (n = 39, 43%) and 60m (n = 10, 11%). Most studies measuring speed used electronic timing gates (n = 82, 91%).

Studies measured cardiovascular fitness in several different forms (n = 87, 35%). Continuous running tests were the most common (n = 48, 55%), primarily via the multi-stage fitness test (n = 44, 92%). Intermittent running assessments were also prevalent (n = 39, 45%), measured predominately through Yo-Yo intermittent recovery test variations (n = 26, 67%) or the 30:15 Intermittent Fitness Test (n = 8, 21%). A minority of studies also assessed players’ repeated sprint ability (n = 10, 11%), through a variety of repeated sprint protocols. Anaerobic fitness testing was also evident in the literature (n = 7, 8%), however no prominent method for measuring this was evident.

Studies measuring muscular strength (n = 83, 34%) were broadly split into those assessing upper body (n = 47, 57%), lower body (n = 58, 70%), and whole-body muscular strength (n = 13, 16%). The number of studies assessing muscular strength was 85% higher in senior players compared to youth players, however these studies still accounted for a similar proportion of total studies assessing physical factors in each cohort (senior = 36%, youth = 34%). The most common measure of lower body muscular strength was the back squat (n = 48, 83%), with these studies normally recording one- (n = 31, 60%) and three-repetition maximums (n = 16, 33%). The bench press was the most common method for measuring upper body muscular strength (n = 43, 91%), also primarily utilising measures of one- (n = 30, 70%) and three-repetition maximums (n = 10, 23%). A limited number of studies also measured upper body strength through the bench pull (n = 7, 15%). Whole body strength assessments were carried out solely through the isometric mid-thigh pull, either using force plates (n = 9, 69%) or a dynamometer (n = 6, 46%).

Agility was assessed in 53 studies (21%), through both pre-planned change of direction assessments (n = 50, 94%) and reactive agility assessments (n = 8, 15%). The most common pre-planned change of direction assessments were the Agility 505 test (n = 26, 52%) and the Agility L-run (n = 11, 22%), which both exclusively reported test duration as a measure of change of direction ability. Studies measuring reactive agility all utilised specific tests of reactive agility designed by the research team, reporting more diverse variables such as response time, decision time, and response and decision accuracy.

Assessments of physical factors involving running such as sprint speed, cardiovascular fitness, and agility were proportionally more common in youth players compared to senior players. In studies assessing physical factors in youth players, 50% assessed sprint speed, 48% assessed cardiovascular fitness, and 32% assessed agility, in contrast to 27% assessing sprint speed, 27% assessing cardiovascular fitness, and 16% assessing agility in senior players.

#### Health factors.

Injury was the most prevalent health factor (n = 90, 68%), normally in the form of rate of injury (n = 74, 82%) captured through medical reports. The number of studies assessing injuries was nearly three times higher in senior players (n = 74) compared to youths (n = 25), with these studies accounting for 74% of all health-based studies in seniors compared to 64% in youth players. Nine injury studies also recorded players’ concussion history (10%) and eight studies measured players’ cognitive performance in relation to assessing concussion (11%). Assessments of cognitive performance usually consisted of assessing reading, memory, and comprehension such as the Digit Symbol Substitution Test, Symbol Digit Modalities Test, and Speed of Comprehension Test.

A sub-section of studies assessing health-related factors focused on more lifestyle-related factors such as sleep (n = 14, 11%) and diet (n = 14, 11%). Of these studies, only one assessed sleep in youth players, compared to 11 in senior players. Sleep was most often monitored via assessment of sleep quality (n = 9, 64%; e.g., sleep efficiency, sleep onset latency), sleep quantity (n = 9, 64%), and sleep patterns (n = 8, 57%; e.g., bedtime, awake time). Studies monitoring sleep employed a mix of methods, including wrist actigraphy devices (n = 7, 43%) and sleep diaries (n = 4, 21%), in addition to a range of self-report questionnaires (e.g., Athlete Sleep Behaviour Questionnaire, Karolinska Sleepiness Scale, Epworth Sleepiness Scale). Studies assessing dietary factors generally tracked intakes of specific nutrients (e.g., carbohydrates, fats, protein), in addition to total energy intake (n = 8, 57%) and total energy expenditure (n = 5, 36%). Methods for monitoring dietary factors varied between convenience methods such as food diaries (n = 4, 29%) and more advanced methods such as doubly labelled water (n = 4, 29%).

Fourteen studies assessed athlete wellness (11%), all through self-report questionnaires. Fatigue was also assessed in a health context in three studies (3%), through subjective measures such as perceived recovery and perceived fatigue, also using self-report questionnaires. Studies assessing fatigue but classified under the physical higher order theme (n = 36, 15%), used direct physical measures such as blood biomarkers (n = 23, 64%; e.g., creatine kinase and blood lactate) and neuromuscular fatigue (n = 22, 61%). Countermovement jumps (n = 17, 73%) and plyometric push-ups (n = 5, 23%) were the most common methods for monitoring neuromuscular fatigue.

#### Technical-tactical factors.

Defensive and offensive involvements represent factors quantifying the occurrence of specific technical actions, such as number of tackles or carries, without assessing the technical execution of these skills. Considering the setting that defensive and offensive involvements were recorded in; 29 of the 32 studies recorded defensive or offensive involvements from match play (91%), two from small-sided games (6%), and one from a training drill (3%). All these studies used video analysis to quantify technical involvements. The most common defensive involvement assessed was tackling (n = 21, 81%), followed by defensive errors (n = 4, 15%). Of the 26 studies recording offensive involvements (45%), 17 recorded number of carries (65%), making this the most common offensive involvement. Eight studies also assessed the number of offensive errors (31%), five studies assessed passing (19%), whilst a range of other offensive involvements featured in three studies each (12%) including offloads, support runs, play the balls, and kicks. Defensive and offensive involvements were more commonly assessed in senior players (defensive involvements n = 24 46%; offensive involvements n = 25, 48%) compared to youth players (defensive involvements n = 4, 31%; offensive involvements n = 5, 38%) and represented a greater proportion of studies assessing technical-tactical factors in senior players.

Defensive and offensive skill variables represent studies assessing technical proficiency, (e.g., subjective tackling proficiency or execution of 2v1 situations), rather than simply the number of times a technical action occurs. In the 24 studies assessing defensive skills (44%), the most common skill was the 1v1 tackle (n = 18, 75%), normally assessed within a standardised drill (n = 16; 89%). Four studies also used a combination of coaches’ subjective judgement alongside video analysis (22%). In each instance video analysis was used to calculate the tackler’s velocity into contact, in addition to coaches subjectively assessing tackle proficiency. Four studies assessed tackling based on specific tackle types (17%) rather than general 1v1 tackling (e.g., head on, rear, over the ball, side on and under the ball tackling). These were all assessed based on coaches’ subjective rating of pre-established criteria, from a mixture of match-play and training drills.

Offensive skills were less commonly assessed than defensive skills (n = 11). Four of these studies assessed general passing skills (36%), primarily through coaches’ subjective rating of pre-established criteria (n = 3, 50%). One study also used video analysis to assess variables related to the technical execution of passes, such as pass duration and pass accuracy. Technical execution of 2v1 situations was another common offensive skill in the literature (n = 7, 50%); all these studies relied on coaches’ subjective rating of pre-established criteria. These assessments were conducted in matches, drills, and small-sided games. One study also assessed execution of 3v2 and 4v3 situations. Discipline, in terms of the number of times players made errors or were penalised were also assessed in four studies (10%), whilst two studies assessed a player’s general skills (3%) by using summative scores for a range of technical skills. The number of studies assessing offensive and defensive skills was lower in youth players (defensive skills n = 5, 38%; offensive skills n = 3, 23%) compared to senior players (defensive skills n = 21, 40%; offensive skills n = 9, 17%) but represented a greater proportion of total studies assessing technical-tactical factors in youth players.

#### Psychological factors.

Research profiling psychological factors predominantly monitored mental health (n = 13, 52%) and psychological skills and characteristics (n = 8, 32%). Studies assessing mental health mostly focused on stress (n = 9, 69%), depression (n = 5, 38%) and anxiety (n = 4, 31%). These factors were assessed using a range of different questionnaires such as the Depression, Anxiety and Stress Scale (21 item) or the 10-item Perceived Stress Scale. Studies focusing on psychological skills and characteristics assessed mental toughness (n = 2, 25%), mental resilience (n = 2, 25%), hardiness (n = 1, 13%) and self-efficacy (n = 1, 13%), through a range of self-report questionnaires such as the Mental Toughness Questionnaire 48 and the Connor-Davidson Resilience Scale. Brain function was assessed exclusively through cognitive performance (n = 5, 100%), normally using video-based tests of pattern recall and prediction (n = 3, 60%). Only three studies were identified which assessed psychological factors in youth players, which collectively measured mental health (n = 2), psychological skills and characteristics (n = 2), and personality traits (n = 1).

#### Other.

The most common factor recorded within the ‘other’ category was playing experience (n=42, 70%), usually via the number of years players had spent playing at a specific level or the number of appearances the player had made. Various factors related to players’ social backgrounds were also recorded, including their level of education (n = 4, 7%), ethnicity (n = 4, 7%), parental status (n = 1, 2%), and marital status (n = 1, 2%). Some studies also sought to investigate players’ perspectives and experiences on different matters. One study discussed players’ role models through questionnaires, with questions relating to who their role models were and their reasons for choosing them. Two studies also recorded players’ lived experiences of progressing through a professional rugby league pathway. These studies used interviews to understand being part of a professional pathway and subsequently transitioning into first team environments from the perspective of the player, including challenges and barriers they had experienced.

#### Supplementary material.

The results above provide an overview of the most common factors and methods identified in the systematic scoping review. Due to the scale of the systematic scoping review, it is beyond the scope of this paper to discuss all the data generated, therefore a more comprehensive overview of factors identified in the systematic scoping review are available in S4 File.

### Part 2: Delphi consensus

#### Round 1.

Participants initially identified 120 different individual factors which they believed should be included as part of the multidimensional profile of a rugby league player. These factors were categorised into five higher order themes: physical, psychological, technical-tactical, health-related and player information. Of the 120 factors suggested, 28 were physical, 34 were psychological, 22 were technical-tactical, 23 were health-related and 13 were categorised under player information.

The median number of methods suggested per factor was two for physical, psychological, technical-tactical, and health-related factors, and one for player information factors. Two physical, one health-related, and three player information factors had no methods suggested initially. All technical-tactical and psychological factors had at least one method suggested. Lower body power had eight methods suggested to assess it, the highest number of any factor.

#### Round 2.

Of the original 120 factors identified from Round 1, 77 reached consensus agreement in Round 2. Twenty of the 28 physical factors reached consensus agreement, with a mean consensus level of 82.9 ± 8.0% agreement. Eighteen of 22 psychological factors reached consensus agreement with a mean consensus level of 83.0 ± 6.3%. Eighteen of 22 technical-tactical factors also reached consensus agreement, with a mean consensus level of 84.9 ± 8.4%. Fifteen of 23 health-related factors reached consensus, with a mean consensus level of 83.6 ± 7.0%. Six of 13 player information factors reached consensus, with a mean consensus level of 79.5 ± 6.7%.

Of the 20 physical factors to reach consensus agreement, 11 had one or more of their corresponding methods reach consensus agreement. Six of the 18 psychological factors had one or more methods reach consensus agreement. Seventeen of the 18 technical-tactical factors to reach consensus agreement in Round 2 had one or more methods reach consensus agreement, compared to 11 of the 15 health-related factors and three of the six player information factors that reached consensus agreement.

#### Round 3.

Voting from Round 3 resulted in two additional physical, four psychological and two technical-tactical factors reaching consensus agreement. No additional health-related or player information factors reached consensus agreement in Round 3. Overall, 82% of physical factors reached consensus agreement, in addition to 65% of psychological factors, 91% of technical-tactical, 65% of health-related, and 46% of player information factors.

Following suggestions from members of the expert panel, new methods were also included in the Round 3 questionnaire; ‘player interview’ was included as a method for psychological factors and ‘coach video analysis based on pre-established criteria’ was included as a method for technical-tactical factors. ‘Player interview’ reached consensus for 16 of the psychological factors, whilst ‘coach video analysis against on pre-established criteria’ reached consensus for 10 of the technical-tactical factors.

Over both rounds of voting the mean level of consensus for physical factors decreased slightly from Round 2 to 81.3 ± 8.4% agreement. The overall mean level of consensus for psychological factors and technical-tactical factors also decreased slightly from Round 2 to 81.2 ± 7.0% and 84.0 ± 8.5% respectively.

Four physical factors reached greater than 90% consensus agreement: upper and lower body strength (both 96%), lower body power (93%), and sprint speed (92%). Four psychological factors also achieved greater than 90% consensus agreement: emotional management and mental health (both 92%), and commitment and willingness to learn (both 91%). Eight technical-tactical factors reached consensus agreement above 90%: decision-making ability and passing ability (both 96%), tackle selection ability (95%), ability to execute skills under pressure, ball carrying, and catching (all 92%), and 1v1 tackle technique and play the ball technique (both 91%). Two health-related factors reached greater than 90% consensus agreement: heart health (96%) and injury history (92%). Cognitive function also achieved 90% consensus agreement. No player information factors achieved greater than 90% consensus agreement, the highest level being 88% for playing history.

In Round 3, five physical factors that had already reached consensus agreement had additional methods reach consensus agreement. None of the three new physical factors to reach consensus agreement had additional methods reach consensus agreement. Three new psychological factors reached consensus agreement in Round 3 that had one or more associated methods reach consensus agreement. In addition, 11 psychological factors that reached consensus agreement in Round 2 had additional methods reach consensus agreement in Round 3. One technical-tactical factor to reach consensus agreement in Round 3 had an associated method also reach consensus agreement, whilst seven factors to reach consensus agreement in Round 2 had additional methods reach consensus agreement in Round 3. One health-related and one player information factor that had reached consensus agreement in Round 2 had methods reach consensus agreement in Round 3.

All factors and associated methods to reach consensus agreement over the three rounds are listed in Table 5, alongside the level of consensus reached (percentage agreement).

**Table 5:** Summary of factors and associated methods to reach consensus and their respective levels of agreement.

Category	Factor	% Agreement	Methods	% Agreement
Physical	Lower body strength	96	1-5RM squat	78
			Isometric mid-thigh pull	75
			Force-velocity profiling	70
	Upper body strength	96	1-5RM bench press	81
			1-5RM bench pull	80
	Lower body power	93	Countermovement jump	86
			Single-leg countermovement jump	84
			Force-velocity profiling	80
			Triple-hop	74
	Sprint speed	92	GPS-derived maximum velocity	86
			10m sprint – timing gates	83
			30m sprint – timing gates	74
			20m sprint – timing gates	70
	Intermittent running ability	88	30:15 intermittent fitness test	75
			Yo-Yo test variations	70
	Continuous running ability	88		
	Upper body power	88		
	Acceleration	85	10m sprint – timing gates	71
	Biological maturation status	85	Peak height velocity estimation	82
	Movement competency	83		
	Match-based running volume	81	GPS monitoring	91
	Repeated sprint ability	81		
	Landing mechanics	80	Force plate analysis	86
	Reactive agility	76		
	Training-based running volume	74	GPS monitoring	95
	Anaerobic fitness	73		
	Body mass	73		
	Height	73		
	Physical fatigue	72	Self-report questionnaire	70
	Range of movement	72	Knee to wall test	77
	Adductor strength	70	GroinBar isometric test	84
			Adductor squeeze pressure cuff test	75
	Change of direction	70	Agility 505 test	78
Psychological	Emotional management	92	Player interview	77
	Mental health	92	Player interview	78
			Psychologist assessment	72
			Psychological Skills for Developing Excellence Questionnaire-2	71
	Commitment	91		
	Willingness to learn	91	Player interview	72
	Adaptability	88		
	Communication	88	Coach subjective assessment	74
			Player interview	71
	Competitiveness	86	Coach subjective assessment	78
	Goal setting	83	Player interview	86
	Autonomy	82	Player interview	82
	Leadership	80	Coach subjective assessment	81
			Player interview	76
	Confidence	79		
	Psychological skills	79	Player interview	73
			Psychological Skills for Developing Excellence Questionnaire-2	73
	Cognitive processes	78	Player interview	80
	Focus	78		
	Psychological resilience	78	Player interview	76
			Coach subjective assessment of specifically designed training drills	70
	Self-awareness	78	Coach subjective assessment	81
			Self-report questionnaire	76
			Player interview	76
	Social and emotional development	76	Player interview	76
	Motivation	75	Player interview	84
			Self-determination questionnaire	77
	Anxiety	74	Competitive State Anxiety Inventory-2	79
			Player interview	76
	Mental fatigue	72		
	Professionalism	72	Player interview	75
			Coach subjective assessment	70
	Mental imagery	71	Player interview	83
			Psychological Skills for Developing Excellence Questionnaire-2	80
			The Sport Imagery Ability Questionnaire	73
			Bull’s Mental Skills Questionnaire	73
Technical-Tactical	Decision-making ability	96	Coach video analysis against pre-established criteria	82
			Coach subjective assessment of specifically designed drills	75
	Passing ability	96	Coach video analysis against pre-established criteria	84
			Coach video analysis of matches	83
	Tackle selection ability	95	Coach video analysis of matches	90
	Ability to execute skills under pressure	92	Specific drills designed to stress technical-tactical skills under pressure	71
	Ball carrying	92	Coach video analysis of matches	83
			Grading of technical execution	70
	Catching	92	Coach video analysis of matches	83
			Grading of technical execution	71
	1v1 tackle technique	91	Grading of technical execution	75
			1v1 tackle drills	71
	Play the ball technique	91	Coach video analysis against pre-established technical criteria	88
			Coach video analysis of matches	81
	Tactical execution	87	Coach video analysis against pre-established technical criteria	78
			Coach video analysis of matches	78
	Tactical knowledge	87	Observation during video review sessions	82
			Player interview	77
			Coach subjective assessment	76
	Offloading ability	82	Coach video analysis against pre-established technical criteria	82
			Coach video analysis of matches	77
	Combination tackle technique	78	Coach video analysis of matches	82
			Grading of technical execution	70
	Number of defensive involvements	78	Count of involvements through video analysis	73
	Knowledge of the rules	78		
	High ball retrieval ability	77	Coach video analysis against pre-established technical criteria	88
			Coach video analysis of matches	76
	Kicking from hand	77	Coach video analysis against pre-established technical criteria	84
			Coach video analysis of matches	76
	Dummy half pass ability	75	Coach video analysis against pre-established technical criteria	92
			Coach video analysis of matches	79
	Draw and pass ability	73	Coach video analysis against pre-established technical criteria	81
	Wrestle ability	73	Coach video analysis against pre-established technical criteria	84
			Coach video analysis of matches	79
	Number of offensive involvements	70	Count of involvements through video analysis	75
Health	Heart health	96	Echocardiogram	90
	Injury history	92	Assessment by physio/doctor	96
			Self-report questionnaire	83
	Cognitive function	90	Cogstate testing	80
			Cognigram	73
	Sleep habits	89	Self-report questionnaire	76
	Injury incidence	88	Record all missed matches and training through injury	100
			Assessment by physio/doctor pre- and post-training	83
	Nutritional behaviours	88	Dietician’s subjective assessment	80
	Sleep quality	88	Self-report questionnaire	76
	Sleep quantity	84	Self-report questionnaire	77
	Nutritional knowledge	83	Player interview	86
	Pre-menstrual symptoms	82	Self-report questionnaire	81
	Menstruation regularity	78	Self-report questionnaire	94
	Nutrition literacy	78	Setting progressive tasks associated with key nutrition behaviours	86
	Gut health	74		
	Brain health	73		
	Injury risk	71		
Player information	Playing history	88	Self-report questionnaire	77
	Chronological age	85		
	Training history	85	Player interview	73
			Self-report questionnaire	73
	Birth quartile	74		
	Sporting history	74	Self-report questionnaire	77
	Anti-doping awareness	71	Self-report questionnaire	80
			Player interview	79

Some factors had no methods reach consensus across the three rounds; in which case the methods section is blank, GPS – global positioning systems.

## Discussion

This systematic scoping review and consensus study aimed to establish the most common factors and methods for profiling rugby league players and subsequently develop consensus on the factors and methods experts believe should be used when profiling rugby league players. Disparities were evident in the volume of research assessing the higher order themes identified in the systematic scoping review. Overall, 67% of studies assessed players’ physical factors, 35% assessed health-related factors, 16% assessed factors classified under the ‘other’ higher order theme, 16% assessed technical-tactical factors, and 7% assessed psychological factors. The systematic scoping review also highlighted that only 3% of studies featured female players. The Delphi consensus identified 85 factors that experts agreed should be profiled in rugby league players, spanning five broad areas: physical (n = 22), psychological (n = 22), technical-tactical (n = 20), health-related (n = 15) and player information (n = 6). These findings provide further understanding of the factors which constitute multidimensional talent in rugby league, highlighting that practitioners attempting to identify and monitor talent in the sport should consider a broad range of factors across several domains. Results from this study also show that future research should focus on the assessment of psychological and technical-tactical factors due to the disparity between how many of these factors reached consensus and the amount of research conducted in these areas. Further consensus is needed around specific methods for assessing these factors due to the lack of consensus seen in this study.

### Multi-dimensional profiling

The Delphi consensus findings highlight the need to profile rugby league players based on factors from multiple disciplines (i.e., physical, psychological, technical-tactical, health-related, and player information). Despite this, 68% of studies in the systematic scoping review assessed factors from only one higher order theme. Studies from within rugby league [16,389], soccer [15], and Australian Rules football [390] have emphasised the need for, and benefits of, multi-dimensional profiling. This is likely to be particularly relevant in a sport such as rugby league with multiple varying demands. Players require well-developed physical qualities [3], technical-tactical skills [9,104], and psychological skills [386] to perform, whilst also trying to recover from training and matches and avoid injury [3,274]. Further to this, some studies in the systematic scoping review also assessed the relationships between factors from different disciplines. For example, one study investigated the links between fatigue from air travel on players’ sleep, neuromuscular fatigue, and offensive and defensive involvements within a match [248]. Studies of this nature account for the possible interrelationships between the range of factors that can be profiled in rugby league players, leading to a deeper understanding of the player and how to maximise their performance. There is an evident need for greater multi-dimensional profiling within rugby league to understand the players more broadly [16,389], however considering the different factors collectively, rather than in isolation, may lead to more applicable findings in future research [391].

### Sex-differences

The systematic scoping review identified only 11 studies (3%) with female participants. This sex-based disparity in rugby league research is likely a reflection of the existence of fully professional senior men’s leagues in Australia and England and the lack of any fully professional women’s leagues. Despite this, there has been substantial growth in women’s rugby league in Australia and England [24], which has led to a greater focus on the research needs of this population [21,24]. Building on that work, the Delphi consensus process was designed to be inclusive in nature, so that its results are applicable to as many player cohorts as possible, including female players. This is evidenced in two menstruation-related factors reaching consensus in the study (menstruation regularity, pre-menstrual symptoms). Overall, the findings from the Delphi consensus provide guidance for the profiling of female rugby league players both in research and applied environments, however further research is needed in this population to establish population-specific data (e.g., 36,114). This is particularly relevant for technical-tactical and psychological factors, as no studies were identified in the review which assessed these factors in female participants.

### Age-based differences

A larger body of player profiling research has been established in senior players (n = 275) compared to youth players (n = 133) in rugby league. Given evidence for differences in physical [6,7,90] and technical-tactical factors [9] between these cohorts, it is likely that further research is needed specifically in youth players to understand their multidimensional development. It is recommended that the assessment of youth players is conducted in the context of their phase of growth and development, considering factors such as relative age and biological maturation status [128,136], both of which reached consensus in the Delphi. Discrepancies in the proportion of research dedicated to each higher order theme were also more pronounced in youth cohorts, with 72% of all studies assessing physical factors in youths compared to 62% in senior players. Furthermore, studies assessing technical-tactical and psychological factors accounted for only 10% and 2% of total studies in youth players, in contrast to 19% and 7% in senior players respectively. The lack of multi-dimensional player profiling was also more evident in youth players, with three studies assessing three or more higher order themes in youth players, as opposed to 23 in senior players. It is suggested that focusing on the holistic development of youth players can encourage more effective talent development [392], which should be supported through multidimensional player profiling [393]. Despite this, it appears that further work is needed to establish a base of research on technical-tactical and psychological factors in youth players prior to increasing the amount of multidimensional research taking place. Understanding the longitudinal development of these factors and how this aligns with biological maturation and relative age may be key to using this research to inform effective talent identification and development practices in rugby league and facilitate the holistic development of youth players.

### Physical factors

Studies assessing physical factors were the most common within the systematic scoping review (n = 267; 67%). The established nature of this research base was reflected in the findings from the Delphi consensus, in which all physical factors to reach consensus agreement were also identified in the systematic scoping review. Furthermore, only one physical method (GroinBar; adductor strength) reached consensus agreement that was not identified in the systematic scoping review. This may be a result of the manufacturer-specific nature of the method itself, however this assessment has been used previously to assess adductor strength in Australian Rules football players [394]. The physical factors to reach the greatest levels of consensus agreement in the Delphi (lower and upper body strength; 96%, lower body power; 93%, sprint speed; 92%) were also some of the most commonly assessed factors in the systematic scoping review. This indicates an alignment between expert opinion on the profiling of physical factors for rugby league and research in this area. The specific physical factors assessed varied somewhat depending on the age of the sample. Assessments of running-based factors such as sprint speed, cardiovascular fitness, and agility were proportionally more common in youth players compared to senior players. This may result from youth players’ familiarity with, and competence in, field-based assessments over typically gym-based assessments of muscular strength and power [235]. Despite this, physical factors were still the most commonly assessed higher order theme in youth players, more so than in senior players, suggesting that future research should be directed towards assessing a wider range of multidimensional factors rather than physical factors in isolation [11,393].

Suggestions for methods to profile players’ physical factors were mostly specific in nature, aside from ‘self-report questionnaire’ reaching consensus agreement to monitor physical fatigue and ‘peak height velocity estimation’ for assessing biological maturation status. Methods mostly referred to specific tests (e.g., 30:15 intermittent fitness test; [395]) or pieces of equipment to use (e.g., force plate analysis [7]), likely reflecting the objective nature of physical testing [14] and the plethora of academic literature that exists using specific methods to objectively assess these factors [396]. These methods are often assessed for reliability and validity (e.g., 397,398), and normative data provided to aid practitioners in their choice of method and conducting their own assessments (e.g., 153,398).

### Health-related factors

Studies monitoring players’ health-related factors were the second most prevalent in the systematic scoping review (n = 134; 36%). Whilst these factors were generally physical in nature (e.g., injury), they were focused on players’ physical well-being and medical condition, rather than performance *per se*. Injury was the most common specific health factor identified in the systematic scoping review (n = 90). This was reflected in the Delphi consensus findings where two injury-related factors reached consensus with high levels of agreement: injury history (92%) and injury incidence (88%). This is consistent with studies in the systematic scoping review which monitored injuries, in which 82% of studies recorded injury rate. Injury risk also reached consensus, with only 71% agreement. This level of agreement may reflect the challenges associated with quantifying injury risk; primarily the myriad factors which may relate to players’ injury susceptibility [24,107,302]. Brain health and cognitive function also both reached consensus, reflecting the current emphasis on concussion in sport [399], and rugby league specifically [400–402]. Consensus could not be reached on a method to monitor brain health; however functional magnetic resonance imaging was just below the threshold for consensus (67%). This may be a result of the number of medical experts (n = 5; 16%) on the panel. Within the systematic scoping review, 16 studies assessed concussions, whether through rate of concussion (n = 7), concussion history (n = 9), or assessing concussion symptoms (n = 1). Cognigrams and CogState testing both reached consensus as methods to monitor cognitive function, however neither were present in the systematic scoping review. Studies typically utilised tests of reading and comprehension such as the Speed of Comprehension Test (n = 3) and the Symbol Digit Modalities Test (n = 3) to assess cognitive function in the context of concussion. This may be a result of the specific nature of the Cognigram and CogState methods as tests used in practice, whereby they may not be available to researchers, or the sensitive nature of the data prevent it from being reported. These findings highlight the potential for investigating the utility of these methods to assess cognitive function in the context of brain injuries in rugby league players, given the sparsity of current research literature identified.

Several nutrition and sleep-related factors reached consensus agreement in the Delphi: gut health, nutrition literacy, nutritional behaviours, nutritional knowledge, and sleep habits, sleep quality, and sleep quantity. Whilst sleep and nutrition-related factors were prevalent in the systematic scoping review, they formed a small minority of health-based research (11% and 10% respectively). The sleep-related factors to reach consensus closely mirrored the research literature, where sleep quality (n = 10), sleep quantity (n = 9), and sleep patterns (n = 8) were the most common factors identified. Each of these factors also reached relatively high levels of agreement (≥84% agreement), compared to the threshold for consensus. Despite this, only one study was identified which assessed sleep-related factors in youth players, highlighting this as a prominent area for future research, particularly given previous evidence showing that youth team-sport athletes typically exhibit poorer sleep quality when compared with individual-sport athletes [403]. Indeed, this information could be used to inform the design of interventions targeted towards the enhancement of sleep quality in youth players to enhance well-being and reduce injury risk [404].

Methods to reach consensus to assess sleep-related factors were not consistent with the systematic scoping review findings. Four specific sleep-related questionnaires were identified in the systematic scoping review, however a non-specific ‘self-report questionnaire’ was the only method to reach consensus for sleep-related factors in the Delphi. Furthermore, the most common method for monitoring sleep-related factors in the literature were wrist actigraphy devices (n = 6), which did not reach consensus in the Delphi despite evidence for their effectiveness as a measurement tool [405]. As sleep has shown links with both sporting performance and recovery from exercise [406], understanding how to monitor sleep in rugby league players may be of benefit to practitioners aiming to maximise performance. The range of available methods and inconsistency in findings between the systematic scoping review and Delphi suggests this is a potential area for development.

The nutrition-related factors to reach consensus generally related to the players’ understanding and competence around nutrition. This contrasts with the findings from the systematic scoping review, where 57% of studies objectively quantified players’ energy intake and 36% quantified energy expenditure. Only one study assessed players’ nutritional knowledge and behaviours [84], highlighting how players with superior nutritional knowledge consumed significantly more fruits and vegetables. Given the relationship between nutrition, health, and sporting performance [407], the profiling of rugby league players’ nutritional knowledge, literacy and behaviours could also be a promising area for future research focus.

### Technical-tactical factors

There was strong consensus on technical-tactical factors in this study. The mean level of consensus for factors which were above the 70% threshold was 84.0%, higher than any other category. Furthermore, more technical-tactical factors exceeded 90% consensus agreement than any other category (n = 8). The value placed on technical-tactical skills in rugby league players has been evidenced in research previously, with coaches identifying several position-specific technical-tactical performance indicators they believed were important to the development of youth players [2]. Certain individual technical-tactical skills such as passing [9] and tackling [8,9] have also been shown to discriminate between playing standards in rugby league, further highlighting their importance. Whilst the importance of technical-tactical factors is not in doubt and clear consensus was achieved for numerous factors, the challenge appears to lie in the systematic measurement of these skills.

The systematic scoping review also highlighted that defensive and offensive skills were more commonly assessed in youth players than skill involvements. This may be due to a focus on skill development in youth players, with these assessments typically involving the subjective assessment of players’ technical proficiency in the execution of skills, rather than simply quantifying the number of skilled actions. Indeed, this approach is encouraged when assessing youth athletes [408], with coach subjective assessments also found to be the most popular method of assessment to reach consensus for assessing technical-tactical skills. However, previous research has suggested that the subjective assessment of technical-tactical factors may not be sensitive to change due to a lack of inter-rater reliability [377], therefore future research should focus on establishing reliable and valid methods for assessing technical-tactical factors in youth players that can allow the longitudinal development of these factors to be monitored. Such assessments have been established in other sports such as soccer [409,410], which encourages the holistic development of youth players, rather than focusing on individual higher order themes in isolation [393].

The methods to reach consensus to measure these factors were generally non-specific, relating to coach subjective assessments of players’ skills based on pre-established criteria. This mirrored the findings from the systematic scoping review where 78% of studies assessing defensive skills did so through standardised drills, with coaches assessing technical execution against pre-established criteria. If the range of skills identified in the Delphi consensus are to be systematically assessed, further work is needed to establish the context in which these skills are measured (i.e., match-based or training drill), the design of these tests, and the criteria used to indicate successful execution. Without consensus around these issues, it is likely that assessment of technical-tactical skills will continue to show varying methods. Tackling was the skill most commonly assessed in the systematic scoping review (n = 18), and as such a standardised tackle analysis framework has been established which can encourage consistency in the assessment of tackling as a skill [411]. Similar frameworks may benefit the assessment of players’ other technical-tactical skills, given the apparent lack of consensus around specific methods to assess them. It is likely that these methods will need to combine the ‘coaches’ eye’ with an objective framework [14,15] to encourage consistency of assessment [377].

### Psychological factors

Psychological factors were the least prevalent within the systematic scoping review, with only 7% of studies assessing these factors in total. The dearth of psychological profiling research was further evident in youth samples whereby only three studies were identified (2%). In contrast to this, 18 psychological factors reached consensus agreement in the Delphi. The value placed on psychological factors in rugby league has been emphasised in previous research where coaches rated their importance above several technical-tactical and physical factors for elite youth players [2]. Despite these findings, relatively little research has been conducted in this area since. This issue is exacerbated by the lack of applied practitioners working in this area in English rugby league, which became apparent through the recruitment process for the Delphi. As such, when interpreting the findings from the psychological factors in the Delphi consensus, it should be considered that only two members of the expert panel worked professionally in sports psychology. Subsequently any contrast between findings from the Delphi consensus and systematic scoping review may be due to the differences in professional backgrounds between those conducting research profiling players psychologically and those that participated in the Delphi. This was particularly evident in the conceptual overlap of some of the factors to reach consensus, such as mental imagery and psychological skills, whereby mental imagery could be considered a psychological skill in of itself [412]. The findings do, however, reflect the opinions of researchers and coaches with extensive experience working in rugby league and may provide an alternative perspective on profiling the psychology of a rugby league player. Nevertheless, a lack of clarity with regards to valid and reliable methods to use exists, due to the non-specificity of suggested methods such as ‘player interview’, ‘coach subjective assessment’, and ‘psychologist assessment’. Similar findings are evident in a study on player profiling in basketball, whereby ‘coach observation’ was found to be the most common method suggested to measure psychological and game intelligence factors, whereas more specific tests were suggested for physical factors [26]. This study also featured a sample of basketball and strength and conditioning coaches, suggesting a lack of participants who specialise in sports psychology may hinder the suggestion of more valid and reliable measures for profiling athletes psychologically.

Mental health was the most common psychological factor identified in the systematic scoping review (n = 13) and reached a high level of consensus agreement in the Delphi (92%). This reflects previous research highlighting a higher prevalence of depression and anxiety symptoms in senior professional rugby league players than in the normal general population [383]. The Psychological Characteristics for Developing Excellence Questionnaire-2 (PCDEQ2) and Competitive Sport Anxiety Invevtory-2 (CSAI-2) reached consensus to monitor mental health and anxiety respectively, mirroring the emphasis in the studies included in the systematic scoping review on utilising questionnaires to assess mental health. The systematic scoping review identified 17 different questionnaires to monitor mental health, however neither of the PCDEQ-2 or CSAI-2 were present. These findings indicate a lack of consistency between academic research practice and expert opinion on how to monitor mental health in rugby league players. This is further emphasised by ‘player interview’ reaching consensus as a method for assessing mental health and numerous other factors. Only one study was identified in the literature which utilised interviews as a method for profiling mental health, where they were used to assess youth players’ experience of stress and their coping mechanisms when transitioning into a high-performance environment [384]. This may indicate a pre-disposition to objectively quantify components of a player’s mental health and psychology rather than understand it subjectively. More qualitative methods for profiling players psychologically could be considered in future research.

Psychological skills and characteristics were another common factor within the psychological profiling literature (n = 8). Mental toughness, mental resilience, hardiness, and self-efficacy were all identified as specific factors, whilst multiple psychological skills and characteristics also reached consensus in the Delphi. Although this is an under-researched topic in rugby league, research exists outside the sport indicating the value of these factors in athlete performance and development, across sports and contexts [412]. Consequently, there may be more benefit in future research developing effective interventions for developing players’ psychological skills and characteristics rather than simply monitoring them, as the skills and characteristics which are important to athletes appear consistent across domains and have been established [412].

### Player information factors

Fewer player information factors from Round 1 reached consensus agreement than in any other category (46%). They also had the lowest mean level of agreement for factors to reach consensus (78.5%). The factors which did reach consensus generally consisted of basic information about a player and their career (e.g., playing history, sporting history). This was consistent with findings from the systematic scoping review, where playing experience was the most frequently recorded factor in the ‘other’ higher order theme (n = 42). Several factors which related to more personal information about the player such as ‘socio-economic status’, ‘family background and support’, and ‘engagement in off-field activities’ were suggested in Round 1 but did not reach consensus agreement. This may indicate apprehension on the part of the panel members to ‘over-monitor’ players, particularly in an applied setting where these factors are not necessarily modifiable, or useful to the design of training [413,414]. Training history and birth quartile reaching consensus reflect existing literature indicating that both factors can relate to the physical development of youth players [139,415], and possibly influence the talent identification process [137], however their utility to senior professional players is not yet evident.

### Research and applied practice contrasts

The findings from this study highlighted several contrasts between research-based practice and expert opinion. Ten members of the Delphi expert panel worked as researchers in some capacity (31%), four of whom also held applied roles, indicating the panel was more heavily weighted towards experts working in applied practice. This imbalance may explain some of the discrepant findings between the systematic scoping review and Delphi consensus, as research and applied practice do not always align [18,416–418]. In some instances, findings from the Delphi contradicted established scientific theory, such as a 10m sprint reaching consensus to measure sprint speed, but the 40m sprint not reaching consensus. The first 10 metres of a sprint is usually categorised as part the acceleration phase [419], with football code athletes typically reaching their maximum velocity after at least 15 metres [420], with one study showing rugby players to reach maximum velocity specifically after 33 metres [421]. Furthermore, when considering sleep-related factors, self-report questionnaires were the only method to reach consensus but wrist actigraphy and ‘sleep labs’ (i.e., polysomnography) did not. Polysomnography is considered the gold-standard in terms of assessing sleep, with wrist actigraphy considered a more practical alternative that is still valid for assessing certain aspects of sleep [422]. Self-reported sleep duration has shown a large positive correlation with wrist-actigraphy derived sleep duration in professional rugby league players, however perceived sleep quality showed a much weaker correlation with wrist-actigraphy derived sleep efficiency [333]. These findings highlight the limitations of using self-reported sleep measures in rugby league players beyond assessing sleep quantity.

Applied practitioners working with youth athletes have previously exhibited inconsistent definitions of key concepts and variable adherence to key principles of applied practice [423], suggesting a misunderstanding of key concepts may cause applied practice to deviate from research findings. Additionally, applied practitioners working in elite soccer have shown limited adherence to an injury prevention program which has been evidenced as effective in reducing hamstring injuries [424]. This, however, appears to have resulted more from contextual challenges rather than a misunderstanding of key concepts per se. Likewise, practitioners working with youth rugby league players have been found to utilise training practices that do not align with their own perceptions of physical qualities deemed most important [425]. Ultimately, applied practitioners may have less awareness of current research evidence, whilst also facing contextual challenges that are not present in research environments [18]. Despite this, the practitioners participating in the Delphi study were very experienced and are likely to have an extensive knowledge of effective applied practice.

Although the opinions of applied practitioners have occasionally contrasted with empirical research findings in this study, they offer an practical perspective on profiling rugby league players. Collectively, these contrasts suggest that collaboration is necessary between applied practitioners and researchers to ensure that applied practitioners are familiar with relevant theory, whilst researchers are able to inform applied practice. It has been suggested that these collaborations can be successful when researchers develop research questions which align with the needs of applied practitioners [18]. Based on the findings from this study, it appears applied practitioners believe several technical-tactical and psychological factors to be important when identifying and monitoring talent in rugby league, however there is limited research evidence investigating these factors to inform their practice. This contrast can guide future research directions and encourage greater integration between research and applied practice.

### Limitations

Overall, the systematic scoping review provides a broad overview of the extent and nature of profiling research conducted in rugby league. It has also highlighted gaps in the research literature profiling rugby league players based on the higher order themes and factors assessed in each study. The Delphi consensus findings have provided direction for future research through garnering expert opinion around factors that should be profiled in rugby league players. Like the systematic scoping review, the Delphi consensus study was intended to be broad in nature, including participants from a range of professional backgrounds and sports, and providing participants with a non-specific brief. This broad, multidisciplinary perspective aimed to make the findings generalisable, providing suggestions for future research and practice across a range of disciplines within rugby league. The breadth of the review and Delphi consensus does, however, limit the specificity and depth of the findings to specific contexts and cohorts. Differences in the quantity and proportion of research conducted in youth and senior players suggest that different factors are more or less relevant to different cohorts within the sport (e.g., senior men vs senior women), whilst methods may have varying levels of feasibility dependent on context and resource [16], therefore future research should focus on understanding how the broad range of factors and methods identified in this study can be applied to specific cohorts.

Whilst the Delphi expert panel was experienced (mean time in current or equivalent role 14.9 years), there were imbalances in the number of participants representing each professional area. Specifically, only two participants worked in sports psychology and two in sports nutrition, in comparison to 10 participants working in academic research and nine working in talent pathways (Table 2). The opinions of the Delphi expert panel represent a broad range of views and perspectives, which can encourage greater validity in any consensus that is reached [28]. Nevertheless, the Delphi consensus is a subjective methodology and the imbalance in the professional backgrounds of participants may have influenced their responses, based on the domain specific knowledge that underpinned their opinions on certain topics. This was partially addressed by providing participants with an ‘outside my area of expertise’ option when voting, however the choice whether to provide an opinion remained with the participant. The multi-disciplinary nature of the panel has been evidenced in the broad range of factors and methods identified through the consensus process (Table 5), which were often outside the findings from the systematic scoping review, building on the information generated in the review.

The systematic scoping review was also not without its limitations. The searches for the review were conducted prior to the commencement of the Delphi study in June 2022, leading to a gap between the searches being conducted and publication. Consequently, recent publications will be missing from the search results. Despite this, the purpose of the review was to outline the frequency that different factors are measured in the research literature relative to each other and to inform the Delphi process, therefore the authors feel that the existing volume of studies included in the review (n = 370) mean that any more recent publications would not meaningfully affect the results of the review. Furthermore, presenting the findings of the systematic scoping review to the Delphi consensus expert panel may create the potential for bias in responses. However, the authors felt this was necessary due to the broad, multidisciplinary nature of the systematic scoping review findings and the Delphi consensus aims.

## Conclusions

This systematic scoping review and consensus study provides an overview of player profiling research in rugby league, and the factors and methods experts believe constitute multidimensional talent in the sport. Studies profiling players’ physical factors made up 67% of total studies, meaning there was a comparative lack of focus on other areas such as technical-tactical (15%) and psychological factors (7%). This issue was further evident in youth cohorts, whereby 72% of studies assessed physical factors, but only 10% and 2% assessed technical-tactical and psychological factors respectively. Sex-specific disparities were also evident, with only 3% of studies featuring female participants. Despite these imbalances in the distribution of player profiling research, physical factors constituted only 26% of the 85 factors to reach consensus overall, with 22 psychological and 20 technical-tactical factors also reaching consensus. This suggests that technical-tactical and psychological factors represent major components of talent in rugby league and therefore future research should assess these factors to further understand multidimensional talent in the sport. There also appears to be a lack of consensus related to specific methods to monitor technical-tactical and psychological factors. Stronger consensus around the most appropriate methods to use in these areas is needed to facilitate future research. Multi-dimensional player profiling has been shown to be beneficial to the talent identification and development process in other sports (e.g., soccer [15]; Australian Rules football [390]), therefore the findings from this study can guide talent identification and monitoring in rugby league, from both an applied and research perspective.

## Supporting information

S1 File PRISMA 2020 checklist. (DOCX)

S2 File PRISMA SCr checklist. (DOCX)

S3 File Pre-Delphi literature review findings. (PDF)

S4 File Literature review summary tables. (DOCX)
