# Supplementary material for: Multidimensional profiling of rugby league players: A systematic scoping review and expert Delphi consensus
Source: PLoS One. 2025 Aug 20;20(8):e0327867. doi: 10.1371/journal.pone.0327867 (PMC12367151; doi:10.1371/journal.pone.0327867)
Supplement: S3 File — (PDF) [file pone.0327867.s003.pdf]

Literature Review Findings

LEEDS BECKETT UNIVERSITY

RUGBY FOOTBALL LEAGUE

Background

Talent in sport is suggested to be multi-dimensional, however recent research indicates player profiling in rugby league focuses too heavily on players' physical qualities.

This review was conducted to summarise the profiling that has taken place in rugby league research to date and objectively assess if any bias exists towards specific areas of profiling. It includes data from 370 studies profiling rugby league players.

The findings highlight WHAT has been measured and HOW it has been measured. It also highlights areas that haven't been researched very heavily in rugby league.

Higher Order Themes

- 67% of all studies measured players' physical qualities
- Only 15% assessed players' technical-tactical skills and 7% assessed psychological factors
- 'Other' consisted mostly of studies recording playing experience and factors which didn't fit a specific category

Higher Order Themes	Number of Studies
Physical	247
Health	134
Other	59
Technical/Tactical	56
Psychological	26

Physical Factors

- Anthropometry consisted mainly of studies measuring body mass (n = 126), height (n = 99) and body composition (n = 79)
- Lower body power (n = 114) was more common than upper body power (n = 47), usually measured with jump variations and bench throws respectively
- 91% of studies measuring speed did so with electronic timing gates over 10m (n = 4), 20m (n = 26), 30m (n = 7) and 40m (n = 39)
- Continuous running tests were the most common form of CV fitness tests (n = 48), usually in the form of the multi-stage fitness test (bleep test; 92%)
- Intermittent running tests also common (n = 39), mostly tested with YoYo test variations (n = 27) and 30:15 IFT (n = 8)
- Lower body strength (n = 58) was more common than upper body (n = 47), usually measured via back squat and bench press respectively
- Agility mostly measured through pre-planned change of direction tests (n = 50) e.g., Agility 505 test (52%), Agility L-run (22%), rather than reactive agility (n = 8)

Specific Factors	Number of Studies
Anthropometry	133
Power	129
Speed	90
Cardiovascular Fitness	87
Strength	83
Agility	53
Fatigue	36
Momentum	12
Strength Endurance	12
Hormonal Status	7
Movement Competency	5
Power Endurance	2
Balance	1

Health Factors

- Studies monitoring injuries focused mostly on injury rates (n = 73), as opposed to brain abnormalities or concussion symptoms
- 8 studies measured cognitive performance in a health context, usually via reading, memory and comprehension tests (e.g., Digit Symbol Substitution Test, Speed of Comprehension Test) as part of concussion testing
- Most studies measuring fatigue in a health context focused on perceived wellness (n = 14), using self-report questionnaires
- Sleep was monitored based on sleep quality (n = 10), quantity (n = 9) and patterns (n = 8), using either wrist actigraphy devices (n = 6) or sleep diaries (n = 3)
- Diet was most commonly monitored through total energy intake (n = 8) and total energy expenditure (n = 5), through a combination of the doubly labelled water method (n = 4) and food diaries (n = 4)

Specific Factors	Number of Studies
Injury	90
Fatigue	18
Sleep	15
Diet	14
Illness	4
Hormonal Status	3
Drugs	2
General Health	2
Hydration	2
Cardiovascular Health	1
Defensive Skills	1
Lifestyle	1

Technical/Tactical Factors

- ‘Involvements’ refer to quantification of a skill involvements e.g., number of tackles, number of carries
- ‘Skills’ refer to assessments of technical proficiency e.g., how well a player executes technical points of a tackle
- Most common defensive involvement was tackling (n = 21), exclusively recorded through video analysis of matches
- Most common offensive involvement was ball carrying (n = 16), with 8 studies also recording offensive errors (n = 8), exclusively recorded through video analysis of match play
- Most common defensive skill was the 1v1 tackle (n = 22), usually measured through coaches’ subjective assessment within a training drill (n = 14)

Specific Factors	Number of Studies
Defensive involvements	26
Defensive skills	23
Offensive involvements	22
Offensive skills	16
Discipline	6
General skills	2

Psychological Factors

- Studies monitoring mental health focused predominantly on stress (n = 9), depression (n = 5) and anxiety (n = 4)
- Mental health monitored using questionnaires e.g., Depression, Anxiety and Stress Scale (21 item) or 10-item Perceived Stress Scale
- Studies measuring cognitive performance were most common within psychological skills and characteristics (n = 5), assessed via video-based pattern recall tests
- Characteristics such as mental toughness (n = 2), mental resilience (n = 2), hardiness (n = 1) and self-efficacy (n = 1) were less common – these were also measured using questionnaires e.g., Connor-Davidson Resilience Scale

Specific Factors	Number of Studies
Mental Health	13
Psychological Skills And Characteristics	12
Fatigue	3
Learning	1
Offensive Skills	1
Personality Traits	1
