# Supplementary material for: Multidimensional profiling of rugby league players: A systematic scoping review and expert Delphi consensus
Source: PLoS One. 2025 Aug 20;20(8):e0327867. doi: 10.1371/journal.pone.0327867 (PMC12367151; doi:10.1371/journal.pone.0327867)
Supplement: S4 File — (DOCX) [file pone.0327867.s004.docx]

Literature Review Summary Tables

The following tables provide a comprehensive list of the general and specific factors identified as part of the literature review, separated by their higher order themes.

# Physical Factors

| **General Factor** | **Specific Factor** | **References** |
| --- | --- | --- |
| Agility | Change of direction | (1-50) |
|  | Reactive agility | (51-53) |
| Anthropometry | Body composition | (3, 5, 6, 8, 13-29, 32, 33, 38, 41-50, 54-98) |
|  | Body mass | (3-8, 11-29, 31-34, 36, 38, 41-50, 54, 57, 59, 61-88, 90-143) |
|  | Height | (3-8, 13-29, 31, 32, 34, 36, 41-45, 47-50, 54, 57, 62, 64, 65, 67, 68, 70-88, 90-92, 94-99, 101-106, 111, 112, 117, 118, 120-125, 127, 129-137, 142, 143) |
|  | Muscle architecture | (144, 145) |
|  | Biological maturation | (3, 42, 44-50, 91, 98) |
| Balance | Single-leg balance | (67) |
| Cardiovascular fitness | Continuous running | (3, 11-19, 21-23, 26-29, 31, 33, 35, 38, 42-50, 58, 59, 64-66, 91, 97, 98, 109, 113, 114, 118, 142, 146-150) |
|  | Anaerobic fitness | (17, 70, 74, 148, 151-153) |
|  | Intermittent running | (2, 6-10, 22, 23, 32, 36, 38, 61, 66, 70, 76, 77, 79, 87, 90, 92-94, 96, 115, 136, 147, 150, 154-165) |
|  | Match-specific fitness | (156) |
|  | Repeated sprint ability | (22, 23, 35, 36, 38, 66, 136, 147, 166, 167) |
| Fatigue | Body composition | (78, 168) |
|  | Body temperature | (78, 169) |
|  | Cardiac activity | (170) |
|  | Enzyme activity | (171) |
|  | Blood bio-markers | (59, 70, 74, 109, 152, 154, 159, 161, 168, 169, 171-183) |
|  | Immune activity | (153, 171) |
|  | Neuromuscular fatigue | (9, 159, 161, 168, 171, 175-191) |
|  | Range of movement | (186, 187) |
|  | Tissue oxygenation | (169) |
| Hormonal status | Hormone levels | (59, 86, 109, 171-173, 178, 189, 192, 193) |
|  | Hormone ratios | (59, 109, 171, 173, 189, 192) |
| Momentum | Aerobic running momentum | (118) |
|  | Intermittent running momentum | (162) |
|  | Jump momentum | (194) |
|  | Sprint momentum | (1, 6, 10, 77, 90, 92-94, 194, 195) |
| Movement competency | Overall movement competency | (62) |
|  | Range of movement | (62, 196) |
|  | Specific movement competency | (34, 36, 62, 117, 196) |
|  | Stability | (62, 196) |
| Power | Core power | (26) |
|  | Lower body power | (1-29, 31, 32, 34-36, 41-50, 59, 61, 63-67, 76, 77, 79, 87, 90-94, 96-98, 101, 109, 110, 114, 115, 117, 118, 124-127, 132-134, 138-143, 145, 146, 155, 167, 195-221) |
|  | Upper body power | (3, 6-10, 20, 26, 34, 42-50, 63, 66, 79, 91, 100, 101, 103, 130, 138-141, 155, 197, 198, 200, 212, 215-217, 222-230) |
|  | Whole body power | (66, 137, 143, 195, 199, 200, 231, 232) |
| Power endurance | Lower body power endurance | (26) |
|  | Upper body power endurance | (33) |
| Speed | Maximal speed capability | (1, 3-38, 41-50, 61, 64-66, 76, 77, 79, 87, 90-94, 96-98, 107, 109-111, 113, 114, 116, 135-137, 142-146, 195, 196, 199, 204, 207, 210, 211, 214, 233) |
|  | Sprint mechanics | (89, 234, 235) |
| Strength | Lower body strength | (1, 4, 5, 33, 61, 63, 66, 79, 84, 86, 88, 90, 92-94, 96, 102, 106, 107, 109-111, 113, 115, 116, 118, 132-134, 137-141, 144, 148, 155, 161, 195, 196, 199-202, 204, 207, 211-217, 233, 236-239) |
|  | Upper body strength | (33, 63, 66, 79, 86, 88, 90, 92-94, 96, 100-103, 106, 109, 110, 113, 115, 118, 123, 130, 138-141, 148, 155, 161, 196-198, 200, 206, 207, 212, 215-217, 224, 225, 227, 236, 239-241) |
|  | Whole body strength | (54, 60, 76, 77, 79, 87, 117, 143, 220, 221, 242-244) |
| Strength endurance | Core endurance | (26, 33) |
|  | Upper body endurance | (2, 26, 66, 97, 103, 110, 113, 115, 148, 155, 245) |

# Technical/Tactical Factors

| **General Factors** | **Specific Factors** | **References** |
| --- | --- | --- |
| Defensive involvements | Defensive decision making | (246, 247) |
|  | Defensive errors | (118, 246-248) |
|  | Kick pressure | (246) |
|  | Kick receive | (249) |
|  | Metres conceded | (250) |
|  | Play the ball | (250, 251) |
|  | Tackling | (22, 138, 139, 142, 176, 181, 185, 212, 215, 249, 251-261) |
|  | Try saves | (246) |
| Defensive skills | 1v1 tackling (head on, rear, side on, over the ball, under the ball | (18, 20, 22-25, 53, 113, 139-141, 215, 216, 261-265) |
|  | Anticipation | (266) |
|  | Defensive shape | (28) |
|  | Defensive speed | (28) |
|  | Defensive space | (28) |
| Discipline | Disciplinary errors | (248-250, 257) |
|  | General errors | (250, 253, 257) |
|  | Technical errors | (118) |
| General skills | Skills under fatigue | (27) |
|  | Technical skill rating | (267) |
| Offensive involvements | 3v2s | (268) |
|  | Carrying | (22, 142, 181, 185, 212, 246, 248-250, 252, 253, 255-257, 259, 260, 269) |
|  | Dummy half carrying | (248, 253, 255, 259) |
|  | Goal kicking | (246, 270) |
|  | Kick receipts | (259) |
|  | Kicking | (248, 253, 259) |
|  | Missed tries | (246) |
|  | Offensive errors | (118, 246-249, 255, 259, 271) |
|  | Offloading | (22, 248, 272) |
|  | Passing | (184, 253, 255, 259, 271) |
|  | Play the ball | (246, 249, 251) |
|  | Support running | (246, 249, 260) |
|  | Try assists | (246) |
| Offensive skills | 4v3s, 3v2s, 2v1s | (22, 23, 27, 28, 53, 273, 274) |
|  | Carrying | (27, 28) |
|  | Catching | (27, 28, 265) |
|  | Offloading | (27, 28) |
|  | Passing | (27, 28, 264, 265) |
|  | Play the ball | (27, 28) |
|  | Playmaking | (275) |

# Health Factors

| **General Factor** | **Specific Factor** | **References** |
| --- | --- | --- |
| Athlete wellness | Perceived wellness | (9, 153, 159, 176, 177, 179-181, 189-191, 211, 276, 277) |
| Cardiovascular health | Blood pressure | (56) |
| Diet | Alcohol intake | (75, 278-280) |
|  | Micronutirent intake | (67, 75, 279) |
|  | Macronutrient intake | (75, 99, 279) |
|  | Nutritional knowledge | (99) |
|  | Resting metabolic rate | (108) |
|  | Supplementation use | (281) |
|  | Total energy expenditure | (80, 108, 279, 282, 283) |
|  | Total energy intake | (67, 75, 80, 86, 108, 152, 279, 284) |
| Fatigue | Perceived fatigue | (185) |
|  | Perceived recovery | (58, 285) |
| General health | Medical history | (278, 280, 281) |
| Hydration | Blood bio-markers | (119) |
|  | Sweat composition | (119) |
|  | Urine composition | (79, 119) |
|  | Water intake | (119) |
| Illness | Illness incidence | (119, 276, 286) |
|  | Immune activity | (186) |
| Injury | Balance | (287) |
|  | Brain abnormalities | (288) |
|  | Cognitive performance | (129, 280, 288-293) |
|  | Concussion education | (294) |
|  | Concussion history | (129, 278, 280, 288-291, 293, 294) |
|  | Concussion symptoms | (295) |
|  | Cortical Excitability | (129) |
|  | Generalised joint hypermobility | (85) |
|  | Injury consequence | (128) |
|  | Injury history | (73, 89, 128, 296, 297) |
|  | Injury rate | (13, 15, 21, 31, 53, 55, 62, 65-67, 73, 84, 85, 89, 104, 112, 114, 120, 121, 146, 158, 254, 258, 260, 261, 269, 277, 281, 285, 293, 295, 298-340) |
|  | Injury risk | (53, 306, 341) |
|  | Lower limb discomfort | (120) |
|  | Posture | (73, 89) |
|  | Range of movement | (89, 123) |
|  | Tendon reflex | (122) |
| Sleep | Jet lag | (186) |
|  | Proneness to sleep | (185, 278, 280, 288) |
|  | Sleep apnea | (56) |
|  | Sleep behaviours | (342) |
|  | Sleep patterns | (56, 105, 342-347) |
|  | Sleep quality | (56, 105, 342-348) |
|  | Sleep quantity | (56, 105, 342-348) |

# Psychological Factors

| **General Factors** | **Specific Factors** | **References** |
| --- | --- | --- |
| Fatigue | Cognitive performance | (168, 175) |
| Mental health | Anger | (349) |
|  | Anxiety | (278, 280, 349, 350) |
|  | Athletic identity | (351, 352) |
|  | Depression | (278, 280, 288, 349, 350) |
|  | Help-seeking behaviour | (352) |
|  | Impulsiveness | (288) |
|  | Mood | (353) |
|  | Overall mental health | (351, 352) |
|  | Pain | (278, 280) |
|  | Perceived cognitive decline | (280, 288) |
|  | Psychological wellbeing | (350, 354) |
|  | Stress | (58, 185, 278, 280, 285, 325, 351, 352, 354) |
| Personality traits | Gratitude | (349) |
|  | Optimism | (349) |
|  | Perseverance | (349) |
|  | Prosocial behaviours | (349) |
| Psychological skills and characteristics | Fine motor control | (129) |
|  | Hardiness | (355) |
|  | Perceptions of psychological skills | (356) |
|  | Self-efficacy | (355) |
|  | Mental toughness | (97, 355) |
|  | Mental resilience | (278, 280) |

# Other Factors

| **General Factors** | **Specific Factors** | **References** |
| --- | --- | --- |
| Playing experience | Playing experience | (2, 11, 12, 14, 17, 19, 20, 22-24, 28, 29, 51, 64, 66, 67, 75, 84, 89, 99, 105, 112, 114, 115, 120, 122, 128, 146, 212, 261, 278, 280, 281, 288, 289, 291, 294, 296, 351, 352, 357, 358) |
| Social background | First club location | (359) |
|  | Ethnicity | (89, 281, 291, 360) |
|  | Country of birth | (349) |
|  | Identity | (361) |
| Age | Chronological age | (50, 102, 143, 294) |
|  | Relative age | (42, 45, 359, 362) |
|  | Resistance training age | (2, 298) |
| Training history | Training history | (90, 298) |
| Marital status | Marital status | (351) |
| Parental status | Parental status | (351) |
| Role models | Role models | (363) |
| Footwear selection | Footwear selection | (120) |
| Lived experience | Player perceptions of club/pathway | (364) |
|  | Player perceptions of transitioning to first-team environment | (365) |
| Equipment use | Equipment use | (89, 281, 296) |
|  | Mouthguard use | (297) |
| Education | Education history | (99, 129, 290, 291) |
| Employment history | Employment history | (291) |
| Preferred handedness | Preferred handedness | (129, 291) |
| Primary language | Primary language | (291) |
| Nationality | Nationality | (362) |

# References

1. Baker D, Newton R. Comparison of Lower Body Strength, Power, Acceleration, Speed, Agility, and Sprint Momentum to Describe and Compare Playing Rank among Professional Rugby League Players. The Journal of Strength & Conditioning Research. 2008;22:153.

2. Booth M, Cobley S, Halaki M, Orr R. Is training age predictive of physiological performance changes in developmental rugby league players? A prospective longitudinal study. International Journal of Sports Science & Coaching. 2020;15(3):306-15.

3. Cobley SP, Till K, O'Hara J, Cooke C, Chapman C. Variable and Changing Trajectories in Youth Athlete Development: Further Verification in Advocating a Long-term Inclusive Tracking Approach. The Journal of Strength & Conditioning Research. 2014;28(7):1959-70.

4. Comfort P, Graham-Smith P, Matthews MJ, Bamber C. Strength and power characteristics in English elite rugby league players. J Strength Cond Res. 2011;25(5):1374-84.

5. Delaney JA, Scott TJ, Ballard DA, Duthie GM, Hickmans JA, Lockie RG, et al. Contributing Factors to Change-of-Direction Ability in Professional Rugby League Players. J Strength Cond Res. 2015;29(10):2688-96.

6. Dobbin N, Gardner A, Daniels M, Twist C. The influence of preseason training phase and training load on body composition and its relationship with physical qualities in professional junior rugby league players. J Sports Sci. 2018;36(24):2778-86.

7. Dobbin N, Highton J, Moss S, Twist C. The Discriminant Validity of Standardised Testing Battery and Its Ability to Differentiate Anthropometric and Physical Characteristics Between Youth, Academy and Senior Professional Rugby League Players. International Journal of Sports Physiology and Performance. 2019;14:1-21.

8. Dobbin N, Highton J, Moss SL, Twist C. Factors Affecting the Anthropometric and Physical Characteristics of Elite Academy Rugby League Players: A Multiclub Study. Int J Sports Physiol Perform. 2019;14(7):958-65.

9. Dobbin N, Highton J, Moss SL, Twist C. The Effects of In-Season, Low-Volume Sprint Interval Training With and Without Sport-Specific Actions on the Physical Characteristics of Elite Academy Rugby League Players. Int J Sports Physiol Perform. 2020;15(5):705-13.

10. Dobbin N, Hunwicks R, Highton J, Twist C. A Reliable Testing Battery for Assessing Physical Qualities of Elite Academy Rugby League Players. Journal of Strength and Conditioning Research. 2017;32:1.

11. Gabbett TJ. Physiological characteristics of junior and senior rugby league players. British Journal of Sports Medicine. 2002;36(5):334-9.

12. Gabbett TJ. Influence of physiological characteristics on selection in a semi-professional first grade rugby league team: a case study. J Sports Sci. 2002;20(5):399-405.

13. Gabbett TJ. Changes in Physiological and Anthropometric Characteristics of Rugby League Players During a Competitive Season. Journal of strength and conditioning research / National Strength & Conditioning Association. 2005;19:400-8.

14. Gabbett TJ. A comparison of physiological and anthropometric characteristics among playing positions in junior rugby league players. Br J Sports Med. 2005;39(9):675-80.

15. Gabbett TJ. Physiological and anthropometric characteristics of junior rugby league players over a competitive season. J Strength Cond Res. 2005;19(4):764-71.

16. Gabbett TJ. A comparison of physiological and anthropometric characteristics among playing positions in sub-elite rugby league players. J Sports Sci. 2006;24(12):1273-80.

17. Gabbett TJ. Physiological and anthropometric characteristics of elite women rugby league players. J Strength Cond Res. 2007;21(3):875-81.

18. Gabbett TJ. Influence of Fatigue on Tackling Technique in Rugby League Players. Journal of Strength & Conditioning Research. 2008;22(2).

19. Gabbett TJ. Physiological and anthropometric characteristics of starters and non-starters in junior rugby league players, aged 13-17 years. J Sports Med Phys Fitness. 2009;49(3):233-9.

20. Gabbett TJ. Physiological and anthropometric correlates of tackling ability in rugby league players. J Strength Cond Res. 2009;23(2):540-8.

21. Gabbett TJ, Domrow N. Relationships between training load, injury, and fitness in sub-elite collision sport athletes. J Sports Sci. 2007;25(13):1507-19.

22. Gabbett TJ, Jenkins D, Abernethy B. Relationships between physiological, anthropometric, and skill qualities and playing performance in professional rugby league players. Journal of sports sciences. 2011;29:1655-64.

23. Gabbett TJ, Jenkins D, Abernethy B. Relative importance of physiological, anthropometric, and skill qualities to team selection in professional rugby league. Journal of sports sciences. 2011;29:1453-61.

24. Gabbett TJ, Jenkins DG, Abernethy B. Physiological and anthropometric correlates of tackling ability in junior elite and subelite rugby league players. J Strength Cond Res. 2010;24(11):2989-95.

25. Gabbett TJ, Jenkins DG, Abernethy B. Correlates of Tackling Ability in High-Performance Rugby League Players. The Journal of Strength & Conditioning Research. 2011;25(1):72-9.

26. Gabbett TJ, Johns J, Riemann M. Performance changes following training in junior rugby league players. J Strength Cond Res. 2008;22(3):910-7.

27. Gabbett TJ, Kelly J, Pezet T. Relationship Between Physical Fitness and Playing Ability in Rugby League Players. Journal of strength and conditioning research / National Strength & Conditioning Association. 2007;21:1126-33.

28. Gabbett TJ, Kelly J, Pezet T. A comparison of fitness and skill among playing positions in sub-elite rugby league players. Journal of Science and Medicine in Sport. 2008;11(6):585-92.

29. Gabbett TJ, Kelly J, Ralph S, Driscoll D. Physiological and anthropometric characteristics of junior elite and sub-elite rugby league players, with special reference to starters and non-starters. J Sci Med Sport. 2009;12(1):215-22.

30. Gabbett TJ, Kelly JN, Sheppard JM. Speed, change of direction speed, and reactive agility of rugby league players. J Strength Cond Res. 2008;22(1):174-81.

31. Inglis PR, Doma K, Deakin GB. The Incidence and Occurrence of Injuries To Junior Rugby League Players in a Tropical Environment. J Hum Kinet. 2019;67:101-10.

32. Jones B, Emmonds S, Hind K, Nicholson G, Rutherford Z, Till K. Physical Qualities of International Female Rugby League Players by Playing Position. J Strength Cond Res. 2016;30(5):1333-40.

33. Meir R, Newton R, Curtis E, Fardell M, Butler B. Physical fitness qualities of professional rugby league football players: determination of positional differences. J Strength Cond Res. 2001;15(4):450-8.

34. Morley D, Pyke D, Till K. An Investigation into the Use of a Movement Assessment Protocol for Under-14 Rugby League Players in a Talent Development Environment. International Journal of Sports Science & Coaching. 2015;10(4):623-36.

35. Nicholls A, Leicht A, Connor J, Halliday A, Doma K. Convergent validity and reliability of a novel repeated agility protocol in junior rugby league players. F1000Res. 2020;9:624.

36. Pearce LA, Sinclair WH, Leicht AS, Woods CT. Physical, Anthropometric, and Athletic Movement Qualities Discriminate Development Level in a Rugby League Talent Pathway. J Strength Cond Res. 2018;32(11):3169-76.

37. Sayers MG. Influence of Test Distance on Change of Direction Speed Test Results. J Strength Cond Res. 2015;29(9):2412-6.

38. Scott TJ, Duthie GM, Delaney JA, Sanctuary CE, Ballard DA, Hickmans JA, et al. The Validity and Contributing Physiological Factors to 30-15 Intermittent Fitness Test Performance in Rugby League. J Strength Cond Res. 2017;31(9):2409-16.

39. Serpell BG, Ford M, Young WB. The development of a new test of agility for rugby league. J Strength Cond Res. 2010;24(12):3270-7.

40. Serpell BG, Young WB, Ford M. Are the perceptual and decision-making components of agility trainable? A preliminary investigation. J Strength Cond Res. 2011;25(5):1240-8.

41. Sinclair J, Edmundson CJ, Metcalfe J, Bottoms L, Atkins S, Bentley I. The Effects of Sprint vs. Resisted Sled-Based Training; an 8-Week in-Season Randomized Control Intervention in Elite Rugby League Players. Int J Environ Res Public Health. 2021;18(17).

42. Till K, Cobley S, Morley D, O’hara J, Chapman C, Cooke C. The influence of age, playing position, anthropometry and fitness on career attainment outcomes in rugby league. Journal of Sports Sciences. 2016;34(13):1240-5.

43. Till K, Cobley S, O' Hara J, Cooke C, Chapman C. Considering maturation status and relative age in the longitudinal evaluation of junior rugby league players. Scandinavian Journal of Medicine & Science in Sports. 2014;24(3):569-76.

44. Till K, Cobley S, O'Hara J, Brightmore A, Cooke C, Chapman C. Using anthropometric and performance characteristics to predict selection in junior UK Rugby League players. J Sci Med Sport. 2011;14(3):264-9.

45. Till K, Cobley S, O'Hara J, Chapman C, Cooke C. Anthropometric, Physiological and Selection Characteristics in High Performance UK Junior Rugby League Players. Talent Development and Excellence. 2010;2:193-207.

46. Till K, Cobley S, O'Hara J, Chapman C, Cooke C. A longitudinal evaluation of anthropometric and fitness characteristics in junior rugby league players considering playing position and selection level. Journal of science and medicine in sport / Sports Medicine Australia. 2012;16.

47. Till K, Cobley S, O'Hara J, Chapman C, Cooke C. An Individualized Longitudinal Approach to Monitoring the Dynamics of Growth and Fitness Development in Adolescent Athletes. The Journal of Strength & Conditioning Research. 2013;27(5):1313-21.

48. Till K, Cobley S, O'Hara J, Morley D, Chapman C, Cooke C. Retrospective analysis of anthropometric and fitness characteristics associated with long-term career progression in Rugby League. J Sci Med Sport. 2015;18(3):310-4.

49. Till K, Jones BL, Cobley S, Morley D, O'Hara J, Chapman C, et al. Identifying Talent in Youth Sport: A Novel Methodology Using Higher-Dimensional Analysis. PLOS ONE. 2016;11(5):e0155047.

50. Till K, Morley D, O'Hara J, Jones B, Chapman C, Beggs C, et al. A retrospective longitudinal analysis of anthropometric and physical qualities that associate with adult career attainment in junior rugby league players. Journal of Science and Medicine in Sport. 2017;20.

51. Gabbett TJ, Abernethy B. Expert-Novice Differences in the Anticipatory Skill of Rugby League Players. Sport, Exercise, and Performance Psychology. 2013;2:138.

52. Gabbett TJ, Benton D. Reactive agility of rugby league players. J Sci Med Sport. 2009;12(1):212-4.

53. Gabbett TJ, Ullah S, Jenkins D, Abernethy B. Skill qualities as risk factors for contact injury in professional rugby league players. Journal of sports sciences. 2012;30(13):1421-7.

54. Atkins SJ. Normalizing expressions of strength in elite rugby league players. J Strength Cond Res. 2004;18(1):53-8.

55. Awwad GEH, Coleman JH, Dunkley CJ, Dewar DC. An Analysis of Knee Injuries in Rugby League: The Experience at the Newcastle Knights Professional Rugby League Team. Sports Med Open. 2019;5(1):33.

56. Caia J, Halson SL, Scott A, Kelly VG. Obstructive sleep apnea in professional rugby league athletes: An exploratory study. J Sci Med Sport. 2020;23(11):1011-5.

57. Cheng HL, O’Connor H, Kay S, Cook R, Parker H, Orr R. Anthropometric characteristics of Australian junior representative rugby league players. Journal of Science and Medicine in Sport. 2014;17(5):546-51.

58. Coutts AJ, Reaburn P. Monitoring changes in rugby league players' perceived stress and recovery during intensified training. Percept Mot Skills. 2008;106(3):904-16.

59. Coutts AJ, Reaburn P, Piva TJ, Rowsell GJ. Monitoring for overreaching in rugby league players. Eur J Appl Physiol. 2007;99(3):313-24.

60. DosʼSantos T, Jones PA, Comfort P, Thomas C. Effect of Different Onset Thresholds on Isometric Midthigh Pull Force-Time Variables. J Strength Cond Res. 2017;31(12):3463-73.

61. Duthie GM, Thornton HR, Delaney JA, McMahon JT, Benton DT. Relationship Between Physical Performance Testing Results and Peak Running Intensity During Professional Rugby League Match Play. J Strength Cond Res. 2020;34(12):3506-13.

62. Dyer CS, Callister R, Sanctuary CE, Snodgrass SJ. Functional Movement Screening and injury risk in elite adolescent rugby league players. International Journal of Sports Science & Coaching. 2019;14(4):498-506.

63. Fernandes JFT, Daniels M, Myler L, Twist C. Influence of Playing Standard on Upper- and Lower-Body Strength, Power, and Velocity Characteristics of Elite Rugby League Players. J Funct Morphol Kinesiol. 2019;4(2).

64. Gabbett TJ. Physiological and anthropometric characteristics of amateur rugby league players. British journal of sports medicine. 2000;34:303-7.

65. Gabbett TJ. Performance changes following a field conditioning program in junior and senior rugby league players. J Strength Cond Res. 2006;20(1):215-21.

66. Gabbett TJ, Ullah S, Finch CF. Identifying risk factors for contact injury in professional rugby league players--application of a frailty model for recurrent injury. J Sci Med Sport. 2012;15(6):496-504.

67. Georgeson E, Weeks B, McLellan C, Beck B. Seasonal change in bone, muscle and fat in professional rugby league players and its relationship to injury: A cohort study. BMJ open. 2012;2.

68. Greene DA, Varley B, Duncan CS, Gabbett TJ. Assessment of body composition in junior representative and first grade rugby league players using dual x-ray absorptiometry. Science and Medicine in Football. 2017;1(3):197-202.

69. Harley JA, Hind K, O'Hara J P. Three-compartment body composition changes in elite rugby league players during a super league season, measured by dual-energy X-ray absorptiometry. J Strength Cond Res. 2011;25(4):1024-9.

70. Holloway KM, Meir RA, Brooks LO, Phillips CJ. The triple-120 meter shuttle test: a sport-specific test for assessing anaerobic endurance fitness in rugby league players. J Strength Cond Res. 2008;22(2):633-9.

71. Jones B, Till K, Barlow M, Lees M, O'Hara JP, Hind K. Anthropometric and Three-Compartment Body Composition Differences between Super League and Championship Rugby League Players: Considerations for the 2015 Season and Beyond. PLoS One. 2015;10(7):e0133188.

72. Jones B, Till K, Roe G, O'Hara J, Lees M, Barlow MJ, et al. Six-year body composition change in male elite senior rugby league players. J Sports Sci. 2018;36(3):266-71.

73. Langdon E, Snodgrass SJ, Young JL, Miller A, Callister R. Posture of rugby league players and its relationship to non-contact lower limb injury: A prospective cohort study. Phys Ther Sport. 2019;40:27-32.

74. Lovell DI, Mason D, Delphinus E, McLellan C. Upper and lower body anaerobic performance of semi-elite Rugby League players. J Sports Med Phys Fitness. 2013;53(5):477-82.

75. Lundy B, O'Connor H, Pelly F, Caterson I. Anthropometric characteristics and competition dietary intakes of professional rugby league players. Int J Sport Nutr Exerc Metab. 2006;16(2):199-213.

76. McCormack S, Jones B, Elliott D, Rotheram D, Till K. Coaches' Assessment of Players Physical Performance: Subjective and Objective Measures are needed when Profiling Players. European journal of sport science. 2021:1-17.

77. McCormack S, Jones B, Scantlebury S, Collins N, Owen C, Till K. Using Principal Component Analysis to Compare the Physical Qualities Between Academy and International Youth Rugby League Players. International Journal of Sports Physiology and Performance. 2021:1-8.

78. Meir R, Brooks L, Shield T. Body weight and tympanic temperature change in professional rugby league players during night and day games: a study in the field. J Strength Cond Res. 2003;17(3):566-72.

79. Minahan C, Newans T, Quinn K, Parsonage J, Buxton S, Bellinger P. Strong, Fast, Fit, Lean, and Safe: A Positional Comparison of Physical and Physiological Qualities Within the 2020 Australian Women's Rugby League Team. J Strength Cond Res. 2021;35(Suppl 2):S11-s9.

80. Morehen JC, Bradley WJ, Clarke J, Twist C, Hambly C, Speakman JR, et al. The Assessment of Total Energy Expenditure During a 14-Day In-Season Period of Professional Rugby League Players Using the Doubly Labelled Water Method. Int J Sport Nutr Exerc Metab. 2016;26(5):464-72.

81. Morehen JC, Clarke J, Batsford J, Highton J, Erskine RM, Morton JP, et al. Development of anthropometric characteristics in professional Rugby League players: Is there too much emphasis on the pre-season period? Eur J Sport Sci. 2020;20(8):1013-22.

82. Morehen JC, Routledge HE, Twist C, Morton JP, Close GL. Position specific differences in the anthropometric characteristics of elite European Super League rugby players. Eur J Sport Sci. 2015;15(6):523-9.

83. Morgan PJ, Callister R. Effects of a preseason intervention on anthropometric characteristics of semiprofessional rugby league players. J Strength Cond Res. 2011;25(2):432-40.

84. O'Connor D. Groin injuries in professional rugby league players: a prospective study. J Sports Sci. 2004;22(7):629-36.

85. Oddy C, Johnson MI, Jones G. The effect of generalised joint hypermobility on rate, risk and frequency of injury in male university-level rugby league players: a prospective cohort study. BMJ Open Sport Exerc Med. 2016;2(1):e000177.

86. Rogerson S, Riches CJ, Jennings C, Weatherby RP, Meir RA, Marshall-Gradisnik SM. The effect of five weeks of Tribulus terrestris supplementation on muscle strength and body composition during preseason training in elite rugby league players. J Strength Cond Res. 2007;21(2):348-53.

87. Scantlebury S, McCormack S, Sawczuk T, Emmonds S, Collins N, Beech J, et al. The anthropometric and physical qualities of women’s rugby league Super League and international players; identifying differences in playing position and level. PLOS ONE. 2022;17:e0249803.

88. Sinclair J, Edmundson C, Bentley I. The efficacy of repetitions-in-reserve vs. traditional percentage-based resistance training: a 4-week pre-season randomized intervention in elite rugby league players. Sport Sciences for Health. 2022;18.

89. Summers KM, Snodgrass SJ, Callister R. Predictors of calf cramping in rugby league. J Strength Cond Res. 2014;28(3):774-83.

90. Till K, Darrall-Jones J, Weakley JJ, Roe GA, Jones BL. The Influence of Training Age on the Annual Development of Physical Qualities Within Academy Rugby League Players. The Journal of Strength & Conditioning Research. 2017;31(8):2110-8.

91. Till K, Jones B. Monitoring anthropometry and fitness using maturity groups within youth rugby league. J Strength Cond Res. 2015;29(3):730-6.

92. Till K, Jones B, Darrall-Jones J, Emmonds S, Cooke C. Longitudinal development of anthropometric and physical characteristics within academy rugby league players. J Strength Cond Res. 2015;29(6):1713-22.

93. Till K, Jones B, Emmonds S, Tester E, Cooke C. Seasonal Changes in Anthropometric and Physical Characteristics Within English Academy Rugby League Players. The Journal of Strength and Conditioning Research. 2014;28:2689-96.

94. Till K, Jones B, Geeson-Brown T. Do physical qualities influence the attainment of professional status within elite 16–19 year old rugby league players? Journal of Science and Medicine in Sport. 2016;19(7):585-9.

95. Till K, Jones B, O'Hara J, Barlow M, Brightmore A, Lees M, et al. Three-Compartment Body Composition in Academy and Senior Rugby League Players. Int J Sports Physiol Perform. 2016;11(2):191-6.

96. Till K, Tester E, Jones B, Emmonds S, Fahey J, Cooke C. Anthropometric and physical characteristics of english academy rugby league players. Journal of strength and conditioning research. 2014;28(2):319-27.

97. Tredrea M, Dascombe B, Sanctuary CE, Scanlan AT. The role of anthropometric, performance and psychological attributes in predicting selection into an elite development programme in older adolescent rugby league players. J Sports Sci. 2017;35(19):1897-903.

98. Waldron M, Worsfold P, Twist C, Lamb K. Changes in anthropometry and performance, and their interrelationships, across three seasons in elite youth rugby league players. J Strength Cond Res. 2014;28(11):3128-36.

99. Alaunyte I, Perry JL, Aubrey T. Nutritional knowledge and eating habits of professional rugby league players: does knowledge translate into practice? J Int Soc Sports Nutr. 2015;12:18.

100. Baker D. Comparison of upper-body strength and power between professional and college-aged rugby league players. J Strength Cond Res. 2001;15(1):30-5.

101. Baker D. Differences in strength and power among junior-high, senior-high, college-aged, and elite professional rugby league players. J Strength Cond Res. 2002;16(4):581-5.

102. Baker DG. Comparison of Strength Levels Between Players From Within the Same Club Who Were Selected vs. Not Selected to Play in the Grand Final of the National Rugby League Competition. J Strength Cond Res. 2017;31(6):1461-7.

103. Baker DG, Newton RU. Discriminative analyses of various upper body tests in professional rugby-league players. Int J Sports Physiol Perform. 2006;1(4):347-60.

104. Booth M, Orr R. Time-Loss Injuries in Sub-Elite and Emerging Rugby League Players. J Sports Sci Med. 2017;16(2):295-301.

105. Caia J, Scott TJ, Halson SL, Kelly VG. The influence of sleep hygiene education on sleep in professional rugby league athletes. Sleep Health. 2018;4(4):364-8.

106. Callaghan DE, Guy JH, Elsworthy N, Kean C. Validity of the PUSH band 2.0 and Speed4lifts to measure velocity during upper and lower body free-weight resistance exercises. J Sports Sci. 2022;40(9):968-75.

107. Comfort P, Haigh A, Matthews MJ. Are changes in maximal squat strength during preseason training reflected in changes in sprint performance in rugby league players? J Strength Cond Res. 2012;26(3):772-6.

108. Costello N, Deighton K, Preston T, Matu J, Rowe J, Jones B. Are professional young rugby league players eating enough? Energy intake, expenditure and balance during a pre-season. Eur J Sport Sci. 2019;19(1):123-32.

109. Coutts A, Reaburn P, Piva TJ, Murphy A. Changes in selected biochemical, muscular strength, power, and endurance measures during deliberate overreaching and tapering in rugby league players. Int J Sports Med. 2007;28(2):116-24.

110. Coutts AJ, Murphy AJ, Dascombe BJ. Effect of direct supervision of a strength coach on measures of muscular strength and power in young rugby league players. J Strength Cond Res. 2004;18(2):316-23.

111. de Lacey J, Brughelli ME, McGuigan MR, Hansen KT. Strength, speed and power characteristics of elite rugby league players. J Strength Cond Res. 2014;28(8):2372-5.

112. Gabbett TJ. Severity and cost of injuries in amateur rugby league: a case study. J Sports Sci. 2001;19(5):341-7.

113. Gabbett TJ. Influence of Fatigue on Tackling Ability in Rugby League Players: Role of Muscular Strength, Endurance, and Aerobic Qualities. PLoS One. 2016;11(10):e0163161.

114. Gabbett TJ, Domrow N. Risk factors for injury in subelite rugby league players. Am J Sports Med. 2005;33(3):428-34.

115. Gabbett TJ, Seibold AJ. Relationship between tests of physical qualities, team selection, and physical match performance in semiprofessional rugby league players. J Strength Cond Res. 2013;27(12):3259-65.

116. Harris NK, Cronin JB, Hopkins WG, Hansen KT. Squat jump training at maximal power loads vs. heavy loads: effect on sprint ability. J Strength Cond Res. 2008;22(6):1742-9.

117. Ireton M, Till K, Weaving D, Jones B. Differences in the Movement Skills and Physical Qualities of Elite Senior & Academy Rugby League Players. The Journal of Strength and Conditioning Research. 2019;33:1.

118. Johnston R. Influence of Physical Characteristics and Match Outcome on Technical Errors During Rugby League Match Play. International Journal of Sports Physiology and Performance. 2019;14:1-21.

119. Jones B, Till K, King R, Gray M, OʼHara J. Are Habitual Hydration Strategies of Female Rugby League Players Sufficient to Maintain Fluid Balance and Blood Sodium Concentration During Training and Match-Play? A Research Note From the Field. J Strength Cond Res. 2016;30(3):875-80.

120. Kinchington MA, Ball KA, Naughton G. Effects of footwear on comfort and injury in professional rugby league. J Sports Sci. 2011;29(13):1407-15.

121. King DA, Gissane C. Injuries in amateur rugby league matches in New Zealand: a comparison between a division 1 and a division 2 premier grade team. Clin J Sport Med. 2009;19(4):277-81.

122. Maurini J, Ohmsen P, Condon G, Pope R, Hing W. National Rugby League athletes and tendon tap reflex assessment: a matched cohort clinical study. BMC Musculoskelet Disord. 2016;17(1):454.

123. McDonough A, Funk L. Can glenohumeral joint isokinetic strength and range of movement predict injury in professional rugby league. Physical Therapy in Sport. 2014;15(2):91-6.

124. McMahon JJ, Jones PA, Comfort P. Comparison of Countermovement Jump-Derived Reactive Strength Index Modified and Underpinning Force-Time Variables Between Super League and Championship Rugby League Players. J Strength Cond Res. 2022;36(1):226-31.

125. McMahon JJ, Jones PA, Suchomel TJ, Lake J, Comfort P. Influence of the Reactive Strength Index Modified on Force- and Power-Time Curves. Int J Sports Physiol Perform. 2018;13(2):220-7.

126. McMahon JJ, Lake JP, Comfort P. Identifying and reporting position-specific countermovement jump outcome and phase characteristics within rugby league. PLoS One. 2022;17(3):e0265999.

127. McMahon JJ, Lake JP, Dos'Santos T, Jones PA, Thomasson ML, Comfort P. Countermovement Jump Standards in Rugby League: What is a "Good" Performance? J Strength Cond Res. 2022;36(6):1691-8.

128. Meir RA, McDonald KN, Russell R. Injury consequences from participation in professional rugby league: a preliminary investigation. Br J Sports Med. 1997;31(2):132-4.

129. Pearce AJ, Rist B, Fraser CL, Cohen A, Maller JJ. Neurophysiological and cognitive impairment following repeated sports concussion injuries in retired professional rugby league players. Brain Inj. 2018;32(4):498-505.

130. Rivière M, Louit L, Strokosch A, Seitz LB. Variable Resistance Training Promotes Greater Strength and Power Adaptations Than Traditional Resistance Training in Elite Youth Rugby League Players. J Strength Cond Res. 2017;31(4):947-55.

131. Sartori S, Whiteley R. Pectoralis major ruptures during rugby league tackling - Case series with implications for tackling technique instruction. J Sci Med Sport. 2019;22(12):1298-303.

132. Scott DJ, Ditroilo M, Marshall P. Effect of Accommodating Resistance on the Postactivation Potentiation Response in Rugby League Players. J Strength Cond Res. 2018;32(9):2510-20.

133. Scott DJ, Ditroilo M, Marshall PA. Complex Training: The Effect of Exercise Selection and Training Status on Postactivation Potentiation in Rugby League Players. The Journal of Strength & Conditioning Research. 2017;31(10):2694-703.

134. Seitz LB, Mina MA, Haff GG. Postactivation Potentiation of Horizontal Jump Performance Across Multiple Sets of a Contrast Protocol. J Strength Cond Res. 2016;30(10):2733-40.

135. Seitz LB, Mina MA, Haff GG. A sled push stimulus potentiates subsequent 20-m sprint performance. J Sci Med Sport. 2017;20(8):781-5.

136. Seitz LB, Rivière M, de Villarreal ES, Haff GG. The athletic performance of elite rugby league players is improved after an 8-week small-sided game training intervention. J Strength Cond Res. 2014;28(4):971-5.

137. Seitz LB, Trajano GS, Haff GG. The back squat and the power clean: elicitation of different degrees of potentiation. Int J Sports Physiol Perform. 2014;9(4):643-9.

138. Speranza M, Gabbett T, Greene D, Johnston R, Townshend A. Relationship Between Two Standardized Tackling Proficiency Tests and Rugby League Match-Play Tackle Performance. International Journal of Sports Physiology and Performance. 2017;13:1-23.

139. Speranza MJ, Gabbett TJ, Johnston RD, Sheppard JM. Relationship Between a Standardized Tackling Proficiency Test and Match-Play Tackle Performance in Semiprofessional Rugby League Players. Int J Sports Physiol Perform. 2015;10(6):754-60.

140. Speranza MJA, Gabbett TJ, Greene DA, Johnston RD, Sheppard JM. Changes in Rugby League Tackling Ability During a Competitive Season: The Relationship With Strength and Power Qualities. J Strength Cond Res. 2017;31(12):3311-8.

141. Speranza MJA, Gabbett TJ, Johnston RD, Sheppard JM. Muscular Strength and Power Correlates of Tackling Ability in Semiprofessional Rugby League Players. Journal of strength and conditioning research. 2015;29(8):2071-8.

142. Waldron M, Worsfold PR, Twist C, Lamb K. The relationship between physical abilities, ball-carrying and tackling among elite youth rugby league players. Journal of Sports Sciences. 2014;32(6):542-9.

143. West DJ, Owen NJ, Jones MR, Bracken RM, Cook CJ, Cunningham DJ, et al. Relationships between force-time characteristics of the isometric midthigh pull and dynamic performance in professional rugby league players. J Strength Cond Res. 2011;25(11):3070-5.

144. McGrath TM, Hulin BT, Pickworth N, Clarke A, Timmins RG. Determinants of hamstring fascicle length in professional rugby league athletes. J Sci Med Sport. 2020;23(5):524-8.

145. Bellinger P, Bourne MN, Duhig S, Lievens E, Kennedy B, Martin A, et al. Relationships between Lower Limb Muscle Characteristics and Force-Velocity Profiles Derived during Sprinting and Jumping. Med Sci Sports Exerc. 2021;53(7):1400-11.

146. Gabbett TJ. Reductions in pre-season training loads reduce training injury rates in rugby league players. Br J Sports Med. 2004;38(6):743-9.

147. Gabbett TJ, Stein JG, Kemp JG, Lorenzen C. Relationship between tests of physical qualities and physical match performance in elite rugby league players. J Strength Cond Res. 2013;27(6):1539-45.

148. Gabbett TJ, Wheeler AJ. Predictors of Repeated High-Intensity-Effort Ability in Rugby League Players. Int J Sports Physiol Perform. 2015;10(6):718-24.

149. Kempton T, Coutts AJ. Factors affecting exercise intensity in professional rugby league match-play. J Sci Med Sport. 2016;19(6):504-8.

150. Scott TJ, McLaren SJ, Caia J, Kelly VG. The reliability and usefulness of an individualised submaximal shuttle run test in elite rugby league players. Science and Medicine in Football. 2018;2(3):184-90.

151. Rovniy A, Pasko V, Martyrosyan A. Adaptation of the cardioresphiratory system to hypoxic actions of the rugby players depending on the playing position. Journal of Physical Education and Sport. 2017;17(2):804-9.

152. Wellington BM, Leveritt MD, Kelly VG. The Effect of Caffeine on Repeat-High-Intensity-Effort Performance in Rugby League Players. Int J Sports Physiol Perform. 2017;12(2):206-10.

153. Partridge EM, Cooke J, McKune AJ, Pyne DB. Partial-Body Cryotherapy Exposure 2 Hours Prior to a Shuttle Run Does Not Enhance Running Performance. Int J Sports Physiol Perform. 2022;17(3):415-22.

154. Atkins SJ. Performance of the Yo-Yo Intermittent Recovery Test by elite professional and semiprofessional rugby league players. J Strength Cond Res. 2006;20(1):222-5.

155. Daniels M, Highton J, Twist C. Pre-season training responses and their associations with training load in elite rugby league players. Science and Medicine in Football. 2019;3(4):313-9.

156. Dobbin N, Highton J, Moss SL, Hunwicks R, Twist C. Concurrent Validity of a Rugby-Specific Yo-Yo Intermittent Recovery Test (Level 1) for Assessing Match-Related Running Performance. J Strength Cond Res. 2021;35(1):176-82.

157. Hulin BT, Gabbett TJ, Johnston RD, Jenkins DG. Sub-maximal heart rate is associated with changes in high-intensity intermittent running ability in professional rugby league players. Science and Medicine in Football. 2019;3(1):50-6.

158. Hulin BT, Gabbett TJ, Pickworth NJ, Johnston RD, Jenkins DG. Relationships Among PlayerLoad, High-Intensity Intermittent Running Ability, and Injury Risk in Professional Rugby League Players. Int J Sports Physiol Perform. 2020;15(3):423-9.

159. Johnston RD, Gabbett TJ, Jenkins DG. Influence of playing standard and physical fitness on activity profiles and post-match fatigue during intensified junior rugby league competition. Sports Med Open. 2015;1(1):18.

160. Johnston RD, Gabbett TJ, Jenkins DG. The Influence of Physical Fitness and Playing Standard on Pacing Strategies During a Team-Sport Tournament. Int J Sports Physiol Perform. 2015;10(8):1001-8.

161. Johnston RD, Gabbett TJ, Jenkins DG, Hulin BT. Influence of physical qualities on post-match fatigue in rugby league players. J Sci Med Sport. 2015;18(2):209-13.

162. Scott TJ, Dascombe BJ, Delaney JA, Sanctuary CE, Scott MTU, Hickmans JA, et al. Running momentum: a new method to quantify prolonged high-intensity intermittent running performance in collision sports. Science and Medicine in Football. 2017;1(3):244-50.

163. Scott TJ, Delaney JA, Duthie GM, Sanctuary CE, Ballard DA, Hickmans JA, et al. Reliability and Usefulness of the 30-15 Intermittent Fitness Test in Rugby League. J Strength Cond Res. 2015;29(7):1985-90.

164. Scott TJ, McLaren SJ, Lovell R, Scott MTU, Barrett S. The reliability, validity and sensitivity of an individualised sub-maximal fitness test in elite rugby league athletes. J Sports Sci. 2022;40(8):840-52.

165. Scott TJ, Thornton HR, Scott MTU, Dascombe BJ, Duthie GM. Differences Between Relative and Absolute Speed and Metabolic Thresholds in Rugby League. Int J Sports Physiol Perform. 2018;13(3):298-304.

166. Johnston RD, Gabbett TJ. Repeated-sprint and effort ability in rugby league players. J Strength Cond Res. 2011;25(10):2789-95.

167. Kilduff LP, West DJ, Williams N, Cook CJ. The influence of passive heat maintenance on lower body power output and repeated sprint performance in professional rugby league players. J Sci Med Sport. 2013;16(5):482-6.

168. Murphy AP, Snape AE, Minett GM, Skein M, Duffield R. The effect of post-match alcohol ingestion on recovery from competitive rugby league matches. J Strength Cond Res. 2013;27(5):1304-12.

169. Selfe J, Alexander J, Costello JT, May K, Garratt N, Atkins S, et al. The effect of three different (-135°C) whole body cryotherapy exposure durations on elite rugby league players. PLoS One. 2014;9(1):e86420.

170. Edmonds RC, Sinclair WH, Leicht AS. Effect of a training week on heart rate variability in elite youth rugby league players. Int J Sports Med. 2013;34(12):1087-92.

171. Roberts LA, Caia J, James LP, Scott TJ, Kelly VG. Effects of External Counterpulsation on Postexercise Recovery in Elite Rugby League Players. Int J Sports Physiol Perform. 2019;14(10):1350-6.

172. McLellan CP, Lovell DI, Gass GC. Biochemical and endocrine responses to impact and collision during elite Rugby League match play. J Strength Cond Res. 2011;25(6):1553-62.

173. McLellan CP, Lovell DI, Gass GC. Creatine kinase and endocrine responses of elite players pre, during, and post rugby league match play. J Strength Cond Res. 2010;24(11):2908-19.

174. Lovell DI, Mason DG, Delphinus EM, McLellan CP. Do compression garments enhance the active recovery process after high-intensity running? J Strength Cond Res. 2011;25(12):3264-8.

175. Skein M, Duffield R, Minett GM, Snape A, Murphy A. The effect of overnight sleep deprivation after competitive rugby league matches on postmatch physiological and perceptual recovery. Int J Sports Physiol Perform. 2013;8(5):556-64.

176. Johnston RD, Gabbett TJ, Jenkins DG. Influence of an intensified competition on fatigue and match performance in junior rugby league players. J Sci Med Sport. 2013;16(5):460-5.

177. Johnston RD, Gabbett TJ, Seibold AJ, Jenkins DG. Influence of physical contact on neuromuscular fatigue and markers of muscle damage following small-sided games. J Sci Med Sport. 2014;17(5):535-40.

178. McLellan CP, Lovell DI, Gass GC. Markers of postmatch fatigue in professional Rugby League players. J Strength Cond Res. 2011;25(4):1030-9.

179. Morehen JC, Clarke J, Batsford J, Barrow S, Brown AD, Stewart CE, et al. Montmorency tart cherry juice does not reduce markers of muscle soreness, function and inflammation following professional male rugby League match-play. Eur J Sport Sci. 2021;21(7):1003-12.

180. Twist C, Waldron M, Highton J, Burt D, Daniels M. Neuromuscular, biochemical and perceptual post-match fatigue in professional rugby league forwards and backs. Journal of Sports Sciences. 2012;30(4):359-67.

181. Johnston RD, Gibson NV, Twist C, Gabbett TJ, MacNay SA, MacFarlane NG. Physiological responses to an intensified period of rugby league competition. J Strength Cond Res. 2013;27(3):643-54.

182. Oxendale CL, Twist C, Daniels M, Highton J. The Relationship Between Match-Play Characteristics of Elite Rugby League and Indirect Markers of Muscle Damage. Int J Sports Physiol Perform. 2016;11(4):515-21.

183. Webb NP, Harris NK, Cronin JB, Walker C. The relative efficacy of three recovery modalities after professional rugby league matches. J Strength Cond Res. 2013;27(9):2449-55.

184. Johnston RD, Gabbett TJ, Jenkins DG, Speranza MJ. The Effect of Different Repeated-High-Intensity-Effort Bouts on Subsequent Running, Skill Performance, and Neuromuscular Function. Int J Sports Physiol Perform. 2016;11(3):311-8.

185. McGuckin TA, Sinclair WH, Sealey RM, Bowman P. The effects of air travel on performance measures of elite Australian rugby league players. Eur J Sport Sci. 2014;14 Suppl 1:S116-22.

186. Fowler PM, Duffield R, Lu D, Hickmans JA, Scott TJ. Effects of Long-Haul Transmeridian Travel on Subjective Jet-Lag and Self-Reported Sleep and Upper Respiratory Symptoms in Professional Rugby League Players. Int J Sports Physiol Perform. 2016;11(7):876-84.

187. Weaving D, Dalton Barron N, Hickmans J, Beggs C, Jones B, Scott T. Latent variable dose–response modelling of external training load measures and musculoskeletal responses in elite rugby league players. Journal of Sports Sciences. 2021;39:1-9.

188. McLellan CP, Lovell DI. Neuromuscular responses to impact and collision during elite rugby league match play. J Strength Cond Res. 2012;26(5):1431-40.

189. McLean BD, Coutts AJ, Kelly V, McGuigan MR, Cormack SJ. Neuromuscular, endocrine, and perceptual fatigue responses during different length between-match microcycles in professional rugby league players. Int J Sports Physiol Perform. 2010;5(3):367-83.

190. Twist C, Highton J, Daniels M, Mill N, Close G. Player Responses to Match and Training Demands During an Intensified Fixture Schedule in Professional Rugby League: A Case Study. Int J Sports Physiol Perform. 2017;12(8):1093-9.

191. Aben HGJ, Hills SP, Higgins D, Cooke CB, Davis D, Jones B, et al. The Reliability of Neuromuscular and Perceptual Measures Used to Profile Recovery, and the Time-Course of such Responses following Academy Rugby League Match-Play. Sports (Basel). 2020;8(5).

192. Beaven CM, Hopkins WG, Hansen KT, Wood MR, Cronin JB, Lowe TE. Dose effect of caffeine on testosterone and cortisol responses to resistance exercise. Int J Sport Nutr Exerc Metab. 2008;18(2):131-41.

193. Crewther BT, Sanctuary CE, Kilduff LP, Carruthers JS, Gaviglio CM, Cook CJ. The workout responses of salivary-free testosterone and cortisol concentrations and their association with the subsequent competition outcomes in professional rugby league. J Strength Cond Res. 2013;27(2):471-6.

194. McMahon J, Lake J, Ripley N, Comfort P. Vertical Jump Testing in Rugby League: A Rationale for Calculating Take-Off Momentum. Journal of applied biomechanics. 2020.

195. Comfort P, Pearson SJ. Scaling--which methods best predict performance? J Strength Cond Res. 2014;28(6):1565-72.

196. Waldron M, Gray A, Worsfold P, Twist C. The Reliability of Functional Movement Screening and In-Season Changes in Physical Function and Performance Among Elite Rugby League Players. J Strength Cond Res. 2016;30(4):910-8.

197. Baker D. A series of studies on the training of high-intensity muscle power in rugby league football players. J Strength Cond Res. 2001;15(2):198-209.

198. Baker D. The effects of an in-season of concurrent training on the maintenance of maximal strength and power in professional and college-aged rugby league football players. J Strength Cond Res. 2001;15(2):172-7.

199. Baker D, Nance S. The Relation Between Running Speed and Measures of Strength and Power in Professional Rugby League Players. The Journal of Strength & Conditioning Research. 1999;13.

200. Baker D, Nance S. The Relation Between Strength and Power in Professional Rugby League Players. The Journal of Strength & Conditioning Research. 1999;13(3):224-9.

201. Baker D, Nance S, Moore M. The load that maximizes the average mechanical power output during jump squats in power-trained athletes. J Strength Cond Res. 2001;15(1):92-7.

202. Brown SR, Brughelli M. Determining return-to-sport status with a multi-component assessment strategy: a case study in rugby. Phys Ther Sport. 2014;15(3):211-5.

203. Callaghan DE, Guy JH, Kean CO, Scanlan AT, Kertesz AHM, Elsworthy N. Back squat velocity to assess neuromuscular status of rugby league players following a match. J Sci Med Sport. 2021;24(1):36-40.

204. Cronin JB, Hansen KT. Strength and power predictors of sports speed. J Strength Cond Res. 2005;19(2):349-57.

205. de Lacey J, Brughelli M, McGuigan M, Hansen K, Samozino P, Morin JB. The effects of tapering on power-force-velocity profiling and jump performance in professional rugby league players. J Strength Cond Res. 2014;28(12):3567-70.

206. Haines MR. Differences in Glenohumeral Joint Rotation and Peak Power Output Between Super League and Championship Rugby League Players. The Journal of Strength & Conditioning Research. 2018;32(6):1685-91.

207. Kirkpatrick J, Comfort P. Strength, power, and speed qualities in English junior elite rugby league players. J Strength Cond Res. 2013;27(9):2414-9.

208. McMahon J, Murphy S, Rej S, Comfort P. Countermovement-Jump-Phase Characteristics of Senior and Academy Rugby League Players. International Journal of Sports Physiology and Performance. 2017;12:803-11.

209. McMahon JJ, Suchomel TJ, Lake JP, Comfort P. Relationship Between Reactive Strength Index Variants in Rugby League Players. J Strength Cond Res. 2021;35(1):280-5.

210. Nicholson B, Dinsdale A, Jones B, Till K. Sprint and Jump Mechanical Profiles in Academy Rugby League Players: Positional Differences and the Associations between Profiles and Sprint Performance. Sports (Basel). 2021;9(7).

211. Orange ST, Metcalfe JW, Robinson A, Applegarth MJ, Liefeith A. Effects of In-Season Velocity- Versus Percentage-Based Training in Academy Rugby League Players. Int J Sports Physiol Perform. 2020;15(4):554-61.

212. Redman KJ, Wade L, Whitley R, Connick MJ, Kelly VG, Beckman EM. The Relationship Between Match Tackle Outcomes and Muscular Strength and Power in Professional Rugby League. J Strength Cond Res. 2021.

213. Seitz LB, de Villarreal ES, Haff GG. The temporal profile of postactivation potentiation is related to strength level. J Strength Cond Res. 2014;28(3):706-15.

214. Simpson A, Waldron M, Cushion E, Tallent J. Optimised force-velocity training during pre-season enhances physical performance in professional rugby league players. J Sports Sci. 2021;39(1):91-100.

215. Speranza M, Gabbett T, Greene D, Johnston R, Townshend A. Tackle characteristics and outcomes in match-play rugby league: the relationship with tackle ability and physical qualities. Science and Medicine in Football. 2017;1:1-7.

216. Speranza MJ, Gabbett TJ, Johnston RD, Sheppard JM. Effect of Strength and Power Training on Tackling Ability in Semiprofessional Rugby League Players. J Strength Cond Res. 2016;30(2):336-43.

217. Speranza MJA, Gabbett TJ, Greene DA, Johnston RD, Townshend AD, O'Farrell B. An Alternative Test of Tackling Ability in Rugby League Players. Int J Sports Physiol Perform. 2018;13(3):347-52.

218. Strokosch A, Louit L, Seitz L, Clarke R, Hughes JD. Impact of Accommodating Resistance in Potentiating Horizontal-Jump Performance in Professional Rugby League Players. Int J Sports Physiol Perform. 2018;13(9):1223-9.

219. Thomasson ML, Comfort P. Occurrence of fatigue during sets of static squat jumps performed at a variety of loads. J Strength Cond Res. 2012;26(3):677-83.

220. Wade J, Fuller J, Devlin P, Doyle T. Lower body peak force but not power is an important discriminator of elite senior rugby league players. Kinesiology. 2020;52:109-14.

221. Wade JA, Fuller JT, Devlin PJ, Doyle TLA. Senior and Junior Rugby League Players Improve Lower-Body Strength and Power Differently During a Rugby League Season. J Strength Cond Res. 2022;36(5):1367-72.

222. Baker D. Acute effect of alternating heavy and light resistances on power output during upper-body complex power training. J Strength Cond Res. 2003;17(3):493-7.

223. Baker D. Acute negative effect of a hypertrophy-oriented training bout on subsequent upper-body power output. J Strength Cond Res. 2003;17(3):527-30.

224. Baker D. 10-Year Changes in Upper Body Strength and Power in Elite Professional Rugby League Players—The Effect of Training Age, Stage, and Content. Journal of strength and conditioning research / National Strength & Conditioning Association. 2013;27:285-92.

225. Baker D, Nance S, Moore M. The load that maximizes the average mechanical power output during explosive bench press throws in highly trained athletes. J Strength Cond Res. 2001;15(1):20-4.

226. Baker D, Newton RU. Acute effect on power output of alternating an agonist and antagonist muscle exercise during complex training. J Strength Cond Res. 2005;19(1):202-5.

227. Baker DG, Newton RU. Adaptations in upper-body maximal strength and power output resulting from long-term resistance training in experienced strength-power athletes. J Strength Cond Res. 2006;20(3):541-6.

228. Baker DG, Newton RU. Effect of kinetically altering a repetition via the use of chain resistance on velocity during the bench press. J Strength Cond Res. 2009;23(7):1941-6.

229. Clark RA, Bryant AL, Humphries B. A comparison of force curve profiles between the bench press and ballistic bench throws. J Strength Cond Res. 2008;22(6):1755-9.

230. Clark RA, Bryant AL, Pua YH. Examining different aspects of functional performance using a variety of bench throw techniques. J Strength Cond Res. 2010;24(10):2755-61.

231. Comfort P, Allen M, Graham-Smith P. Kinetic Comparisons During Variations of the Power Clean. Journal of strength and conditioning research / National Strength & Conditioning Association. 2011;25:3269-73.

232. Comfort P, Allen M, Graham-Smith P. Comparisons of peak ground reaction force and rate of force development during variations of the power clean. J Strength Cond Res. 2011;25(5):1235-9.

233. Comfort P, Bullock N, Pearson SJ. A comparison of maximal squat strength and 5-, 10-, and 20-meter sprint times, in athletes and recreationally trained men. J Strength Cond Res. 2012;26(4):937-40.

234. Caplan N, Rogers R, Parr MK, Hayes PR. The effect of proprioceptive neuromuscular facilitation and static stretch training on running mechanics. J Strength Cond Res. 2009;23(4):1175-80.

235. Bentley I, Sinclair JK, Atkins SJ, Metcalfe J, Edmundson CJ. Effect of Velocity-Based Loading on Acceleration Kinetics and Kinematics During Sled Towing. J Strength Cond Res. 2021;35(4):1030-8.

236. Alonso-Aubin DA, Chulvi-Medrano I, Cortell-Tormo JM, Picón-Martínez M, Rial Rebullido T, Faigenbaum AD. Squat and Bench Press Force-Velocity Profiling in Male and Female Adolescent Rugby Players. J Strength Cond Res. 2021;35(Suppl 1):S44-s50.

237. Charlton PC, Mentiplay BF, Grimaldi A, Pua YH, Clark RA. The reliability of a maximal isometric hip strength and simultaneous surface EMG screening protocol in elite, junior rugby league athletes. J Sci Med Sport. 2017;20(2):139-45.

238. Elsworthy N, Callaghan DE, Scanlan AT, Kertesz AHM, Kean CO, Dascombe BJ, et al. Validity and Reliability of Using Load-Velocity Relationship Profiles to Establish Back Squat 1 m·s-1 Load. J Strength Cond Res. 2021;35(2):340-6.

239. Orange ST, Metcalfe JW, Liefeith A, Marshall P, Madden LA, Fewster CR, et al. Validity and Reliability of a Wearable Inertial Sensor to Measure Velocity and Power in the Back Squat and Bench Press. J Strength Cond Res. 2019;33(9):2398-408.

240. Baker DG, Newton RU. An analysis of the ratio and relationship between upper body pressing and pulling strength. J Strength Cond Res. 2004;18(3):594-8.

241. Haines MR, Fish M, O'Sullivan D. Seasonal changes in glenohumeral joint isokinetic strength in professional rugby league players. Phys Ther Sport. 2019;39:32-7.

242. Dobbin N, Hunwicks R, Jones B, Till K, Highton J, Twist C. Criterion and Construct Validity of an Isometric Midthigh-Pull Dynamometer for Assessing Whole-Body Strength in Professional Rugby League Players. Int J Sports Physiol Perform. 2018;13(2):235-9.

243. Dos'Santos T, Jones P, Kelly J, McMahon J, Comfort P, Thomas C. Effect of Sampling Frequency on Isometric Midthigh-Pull Kinetics. International Journal of Sports Physiology and Performance. 2019;14:1-6.

244. Till K, Morris R, Stokes K, Trewartha G, Twist C, Dobbin N, et al. Validity of an Isometric Midthigh Pull Dynamometer in Male Youth Athletes. The Journal of Strength & Conditioning Research. 2018;32(2):490-3.

245. Baker DG. Ability and validity of three different methods of assessing upper-body strength-endurance to distinguish playing rank in professional rugby league players. J Strength Cond Res. 2009;23(5):1578-82.

246. Wedding C, Woods CT, Sinclair WH, Gomez MA, Leicht AS. Examining the evolution and classification of player position using performance indicators in the National Rugby League during the 2015–2019 seasons. Journal of Science and Medicine in Sport. 2020;23(9):891-6.

247. Wedding CJ, Gomez MA, Woods CT, Sinclair WH, Leicht AS. Effect of match-related contextual factors on positional performance in the national rugby league. International Journal of Sports Science & Coaching. 2023;18(3):832-8.

248. Parmar N, James N, Hearne G, Jones B. Using principal component analysis to develop performance indicators in professional rugby league. International Journal of Performance Analysis in Sport. 2018;18(6):938-49.

249. Wedding C, Woods C, Sinclair W, Gomez M, Leicht A. Exploring the effect of various match factors on team playing styles in the National Rugby League. International Journal of Sports Science & Coaching. 2021;16(4):976-84.

250. Gabbett TJ. Effects of physical, technical, and tactical factors on final ladder position in semiprofessional rugby league. Int J Sports Physiol Perform. 2014;9(4):680-8.

251. Whitehead S, Till K, Jones B, Beggs C, Dalton Barron N, Weaving D. The use of technical-tactical and physical performance indicators to classify between levels of match-play in elite rugby league. Science and Medicine in Football. 2020;5.

252. Gabbett TJ, Hulin BT. Activity and recovery cycles and skill involvements of successful and unsuccessful elite rugby league teams: A longitudinal analysis of evolutionary changes in National Rugby League match-play. J Sports Sci. 2018;36(2):180-90.

253. Woods CT, Robertson S, Sinclair WH, Till K, Pearce L, Leicht AS. A comparison of game-play characteristics between elite youth and senior Australian National Rugby League competitions. Journal of Science and Medicine in Sport. 2018;21(6):626-30.

254. King D, Hume P, Clark T. The effect of player positional groups on the nature of tackles that result in tackle-related injuries in professional rugby league matches. J Sports Med Phys Fitness. 2011;51(3):435-43.

255. Woods CT, Leicht AS, Jones B, Till K. Game-play characteristics differ between the European Super League and the National Rugby League: Implications for coaching and talent recruitment. International Journal of Sports Science & Coaching. 2018;13(6):1171-6.

256. Eggers T, Cross R, Norris D, Wilmot L, Lovell R. Impact of Microcycle Structures on Physical and Technical Outcomes During Professional Rugby League Training and Matches. Int J Sports Physiol Perform. 2022;17(5):755-60.

257. Mullen T, Twist C, Daniels M, Dobbin N, Highton J. Influence of Contextual Factors, Technical Performance, and Movement Demands on the Subjective Task Load Associated With Professional Rugby League Match-Play. Int J Sports Physiol Perform. 2021;16(6):763-71.

258. King D, Hume PA, Clark T. Nature of tackles that result in injury in professional rugby league. Res Sports Med. 2012;20(2):86-104.

259. Woods CT, Robertson S, Sinclair WH, Collier NF. Non-metric multidimensional performance indicator scaling reveals seasonal and team dissimilarity within the National Rugby League. Journal of Science and Medicine in Sport. 2018;21(4):410-5.

260. Gabbett TJ, Jenkins DG, Abernethy B. Physical collisions and injury in professional rugby league match-play. J Sci Med Sport. 2011;14(3):210-5.

261. Gabbett TJ, Ryan P. Tackling Technique, Injury Risk, and Playing Performance in High-Performance Collision Sport Athletes. International Journal of Sports Science & Coaching. 2009;4(4):521-33.

262. Gabbett TJ, Kelly J. Does Fast Defensive Line Speed Influence Tackling Proficiency in Collision Sport Athletes? International Journal of Sports Science & Coaching. 2007;2(4):467-72.

263. Dobbin N, Richardson D, Myler L, Esen O. Effects of a 12% carbohydrate beverage on tackling technique and running performance during rugby league activity: A randomised, placebo-controlled trial. PLoS One. 2022;17(1):e0262443.

264. Pearce LA, Sinclair WH, Leicht AS, Woods CT. Passing and tackling qualities discriminate developmental level in a rugby league talent pathway. International Journal of Performance Analysis in Sport. 2019;19(6):985-98.

265. Waldron M, Worsfold P, Twist C, Lamb K. The reliability of tests for sport-specific skill amongst elite youth rugby league players. European Journal of Sport Science. 2014;14(sup1):S471-S7.

266. Connor JD, Crowther RG, Sinclair WH. Effect of Different Evasion Maneuvers on Anticipation and Visual Behavior in Elite Rugby League Players. Motor Control. 2018;22(1):18-27.

267. Kempton T, Sirotic AC, Cameron M, Coutts AJ. Match-related fatigue reduces physical and technical performance during elite rugby league match-play: a case study. Journal of Sports Sciences. 2013;31(16):1770-80.

268. Pearce LA, Leicht AS, Gómez-Ruano M-Á, Sinclair WH, Woods CT. The type and variation of evasive manoeuvres during an attacking task differ across a rugby league development pathway. International Journal of Performance Analysis in Sport. 2020;20(6):1134-42.

269. Gabbett TJ. Influence of injuries on team playing performance in Rugby League. J Sci Med Sport. 2004;7(3):340-6.

270. Crowe M, O'Connor D. Eye colour and reaction time to visual stimuli in rugby league players. Percept Mot Skills. 2001;93(2):455-60.

271. Gabbett TJ, Abernethy B, Jenkins DG. Influence of field size on the physiological and skill demands of small-sided games in junior and senior rugby league players. J Strength Cond Res. 2012;26(2):487-91.

272. Wheeler WK, Wiseman R, Lyons K. Tactical and technical factors associated with effective ball offloading strategies during the tackle in rugby league. International Journal of Performance Analysis in Sport. 2011;11(2):392-409.

273. Gabbett TJ, Abernethy B. Dual-task assessment of a sporting skill: influence of task complexity and relationship with competitive performances. J Sports Sci. 2012;30(16):1735-45.

274. Gabbett TJ, Wake M, Abernethy B. Use of dual-task methodology for skill assessment and development: Examples from rugby league. Journal of Sports Sciences. 2011;29(1):7-18.

275. Johnston D, Morrison BW. The Application of Naturalistic Decision-Making Techniques to Explore Cue Use in Rugby League Playmakers. Journal of Cognitive Engineering and Decision Making. 2016;10(4):391-410.

276. Thornton HR, Delaney JA, Duthie GM, Scott BR, Chivers WJ, Sanctuary CE, et al. Predicting Self-Reported Illness for Professional Team-Sport Athletes. Int J Sports Physiol Perform. 2016;11(4):543-50.

277. Killen NM, Gabbett TJ, Jenkins DG. Training loads and incidence of injury during the preseason in professional rugby league players. J Strength Cond Res. 2010;24(8):2079-84.

278. Iverson GL, Van Patten R, Terry DP, Levi CR, Gardner AJ. Predictors and Correlates of Depression in Retired Elite Level Rugby League Players. Front Neurol. 2021;12:655746.

279. Tooley E, Bitcon M, Briggs MA, West DJ, Russell M. Estimates of Energy Intake and Expenditure in Professional Rugby League Players. International Journal of Sports Science & Coaching. 2015;10(2-3):551-60.

280. Van Patten R, Iverson GL, Terry DP, Levi CR, Gardner AJ. Predictors and Correlates of Perceived Cognitive Decline in Retired Professional Rugby League Players. Front Neurol. 2021;12:676762.

281. Orr R, Cheng HL. Incidence and characteristics of injuries in elite Australian junior rugby league players. J Sci Med Sport. 2016;19(3):212-7.

282. Costello N, Deighton K, Cummins C, Whitehead S, Preston T, Jones B. Isolated & Combined Wearable Technology Underestimate the Total Energy Expenditure of Professional Young Rugby League Players; A Doubly Labelled Water Validation Study. J Strength Cond Res. 2022;36(12):3398-403.

283. Routledge HE, Bradley WJ, Shepherd SO, Cocks M, Erskine RM, Close GL, et al. Ultrasound Does Not Detect Acute Changes in Glycogen in Vastus Lateralis of Man. Med Sci Sports Exerc. 2019;51(11):2286-93.

284. Costello N, Deighton K, Dalton-Barron N, Whitehead S, Preston T, Jones B. Can a contemporary dietary assessment tool or wearable technology accurately assess the energy intake of professional young rugby league players? A doubly labelled water validation study. Eur J Sport Sci. 2020;20(9):1151-9.

285. King D, Clark T, Kellmann M. Changes in Stress and Recovery as a Result of Participating in a Premier Rugby League Representative Competition. International Journal of Sports Science & Coaching. 2010;5(2):223-37.

286. Chesson L, Deighton K, Whitehead S, Ramírez-López C, Jones B. Incidence, prevalence and consequences of illness in academy rugby league players. J Sci Med Sport. 2020;23(11):1016-20.

287. Gosselin G, Fagan MJ. The effects of cervical muscle fatigue on balance - a study with elite amateur rugby league players. J Sports Sci Med. 2014;13(2):329-37.

288. Stanwell P, Iverson GL, Van Patten R, Castellani RJ, McCrory P, Gardner AJ. Examining for Cavum Septum Pellucidum and Ventricular Enlargement in Retired Elite-Level Rugby League Players. Front Neurol. 2022;13:817709.

289. King D, Gissane C, Clark T. Concussion in amateur rugby league players in New Zealand: A review of player concussion history. New Zealand Journal of Sports Medicine. 2014;40:64-9.

290. Hinton-Bayre AD, Geffen GM, Geffen LB, McFarland KA, Friis P. Concussion in contact sports: reliable change indices of impairment and recovery. J Clin Exp Neuropsychol. 1999;21(1):70-86.

291. Hinton-Bayre AD, Geffen G, McFarland K. Mild head injury and speed of information processing: a prospective study of professional rugby league players. J Clin Exp Neuropsychol. 1997;19(2):275-89.

292. Hinton-Bayre A. Normative Versus Baseline Paradigms for Detecting Neuropsychological Impairment Following Sports-Related Concussion. Brain Impairment. 2015;16:1-10.

293. King D, Clark T, Gissane C. Use of a rapid visual screening tool for the assessment of concussion in amateur rugby league: a pilot study. J Neurol Sci. 2012;320(1-2):16-21.

294. Longworth T, McDonald A, Cunningham C, Khan H, Fitzpatrick J. Do rugby league players under-report concussion symptoms? A cross-sectional study of elite teams based in Australia. BMJ Open Sport Exerc Med. 2021;7(1):e000860.

295. Iverson GL, Van Patten R, Gardner AJ. Examining Whether Onfield Motor Incoordination Is Associated With Worse Performance on the SCAT5 and Slower Clinical Recovery Following Concussion. Front Neurol. 2020;11:620872.

296. Chapman PJ. Orofacial injuries and the use of mouthguards by the 1984 Great Britain Rugby League touring team. Br J Sports Med. 1985;19(1):34-6.

297. Rayner W. Mouthguard Use in Match Play and Training in a Cohort of Professional Rugby League Players. International Journal of Sports Science & Coaching. 2008;3(1):87-93.

298. Booth M, Cobley S, Orr R. Does a higher training age attenuate injury risk in junior elite rugby league players? International Journal of Sports Science & Coaching. 2019;14(6):779-85.

299. Cummins C, King D, Clark T. Injuries in New Zealand amateur rugby league matches by positional groups. New Zealand Journal of Sports Medicine. 2017;44(2):60-4.

300. Fitzpatrick AC, Naylor AS, Myler P, Robertson C. A three-year epidemiological prospective cohort study of rugby league match injuries from the European Super League. J Sci Med Sport. 2018;21(2):160-5.

301. Gabbett TJ. Incidence, site, and nature of injuries in amateur rugby league over three consecutive seasons. Br J Sports Med. 2000;34(2):98-103.

302. Gabbett TJ. Training injuries in rugby league: an evaluation of skill-based conditioning games. J Strength Cond Res. 2002;16(2):236-41.

303. Gabbett TJ. Incidence of injury in semi-professional rugby league players. Br J Sports Med. 2003;37(1):36-43; discussion -4.

304. Gabbett TJ. Influence of training and match intensity on injuries in rugby league. J Sports Sci. 2004;22(5):409-17.

305. Gabbett TJ. Influence of playing position on the site, nature, and cause of rugby league injuries. J Strength Cond Res. 2005;19(4):749-55.

306. Gabbett TJ. Influence of the limited interchange rule on injury rates in sub-elite Rugby League players. J Sci Med Sport. 2005;8(1):111-5.

307. Gabbett TJ. Incidence of injury in junior rugby league players over four competitive seasons. J Sci Med Sport. 2008;11(3):323-8.

308. Gabbett TJ. The development and application of an injury prediction model for noncontact, soft-tissue injuries in elite collision sport athletes. J Strength Cond Res. 2010;24(10):2593-603.

309. Gabbett TJ, Godbolt RJ. Training injuries in professional rugby league. J Strength Cond Res. 2010;24(7):1948-53.

310. Gabbett TJ, Jenkins D, Abernethy B. Physical collisions and injury during professional rugby league skills training. J Sci Med Sport. 2010;13(6):578-83.

311. Gabbett TJ, Jenkins DG. Relationship between training load and injury in professional rugby league players. J Sci Med Sport. 2011;14(3):204-9.

312. Gabbett TJ, Minbashian A, Finch C. Influence of environmental and ground conditions on injury risk in rugby league. J Sci Med Sport. 2007;10(4):211-8.

313. Gabbett TJ, Ullah S. Relationship between running loads and soft-tissue injury in elite team sport athletes. J Strength Cond Res. 2012;26(4):953-60.

314. Gardner AJ, Howell DR, Iverson GL. National Rugby League match scheduling and rate of concussion. Journal of Science and Medicine in Sport. 2019;22(7):780-3.

315. Gardner AJ, Iverson GL, Edwards S, Tucker R. A Case-Control Study of Tackle-Based Head Injury Assessment (HIA) Risk Factors in the National Rugby League. Sports Med Open. 2021;7(1):84.

316. Gardner AJ, Kohler RM, Levi CR, Iverson GL. Usefulness of Video Review of Possible Concussions in National Youth Rugby League. Int J Sports Med. 2017;38(1):71-5.

317. Gibbs N. Injuries in professional rugby league. A three-year prospective study of the South Sydney Professional Rugby League Football Club. Am J Sports Med. 1993;21(5):696-700.

318. Gissane C, Jennings D, Kerr K, White J. Injury rates in rugby league football: impact of change in playing season. Am J Sports Med. 2003;31(6):954-8.

319. Gissane C, Jennings D, White J, Cumine A. Injury in summer rugby league football: the experiences of one club. Br J Sports Med. 1998;32(2):149-52.

320. Gissane C, Jennings DC, Cumine AJ, Stephenson SE, White JA. Differences in the incidence of injury between rugby league forwards and backs. Aust J Sci Med Sport. 1997;29(4):91-4.

321. Gissane C, White J, Kerr K, Jennings S, Jennings D. Health and safety implications of injury in professional rugby league football. Occup Med (Lond). 2003;53(8):512-7.

322. Hinton-Bayre AD, Geffen G, Friis P. Presentation and mechanisms of concussion in professional Rugby League Football. J Sci Med Sport. 2004;7(3):400-4.

323. Hopkinson M, Nicholson G, Weaving D, Hendricks S, Fitzpatrick A, Naylor A, et al. Rugby league ball carrier injuries: The relative importance of tackle characteristics during the European Super League. Eur J Sport Sci. 2022;22(2):269-78.

324. Hulin BT, Gabbett TJ, Caputi P, Lawson DW, Sampson JA. Low chronic workload and the acute:chronic workload ratio are more predictive of injury than between-match recovery time: a two-season prospective cohort study in elite rugby league players. Br J Sports Med. 2016;50(16):1008-12.

325. King D, Clark T, Kellmann M, Hume P. Stress and recovery changes of injured and noninjured amateur representative rugby league players over a competition season. New Zealand Journal of Sports Medicine. 2017;43:57-63.

326. King D, Gabbett T. Injuries in a national women's rugby league tournament: An initial investigation. 2007.

327. King D, Gabbett T. Injuries in the New Zealand semi-professional rugby league competition. New Zealand Journal of Sports Medicine. 2009;36:6-15.

328. King D, Hume P, Milburn P, Gianotti s. Rugby league injuries in New Zealand: Variations in injury claims and costs by ethnicity, gender, age, district, body site, injury type and occupation. 2009;36.

329. King DA, Gabbett TJ. Training injuries in New Zealand amateur rugby league players. Journal of Science and Medicine in Sport. 2008;11(6):562-5.

330. King DA, Hume PA, Milburn P, Gianotti S. Women's rugby league injury claims and costs in New Zealand. Br J Sports Med. 2010;44(14):1016-23.

331. McKinlay A, McLellan T. The mechanism of concussion injury in rugby league. INTERNATIONAL SPORTMED JOURNAL. 2014;15:328-32.

332. Murray NB, Gabbett TJ, Chamari K. Effect of different between-match recovery times on the activity profiles and injury rates of national rugby league players. J Strength Cond Res. 2014;28(12):3476-83.

333. Orr R, Hamidi J, Levy B, Halaki M. Epidemiology of injuries in Australian junior rugby league players. J Sci Med Sport. 2021;24(3):241-6.

334. Phillips LH, Standen PJ, Batt ME. Effects of seasonal change in rugby league on the incidence of injury. Br J Sports Med. 1998;32(2):144-8.

335. Savage J, Hooke C, Orchard J, Parkinson R. The Incidence of Concussion in a Professional Australian Rugby League Team, 1998-2012. J Sports Med (Hindawi Publ Corp). 2013;2013:304576.

336. Stephenson S, Gissane C, Jennings D. Injury in rugby league: a four year prospective survey. Br J Sports Med. 1996;30(4):331-4.

337. Tee JC, Till K, Jones B. Incidence and characteristics of injury in under-19 academy level rugby league match play: A single season prospective cohort study. J Sports Sci. 2019;37(10):1181-8.

338. Thornton HR, Delaney JA, Duthie GM, Dascombe BJ. Importance of Various Training-Load Measures in Injury Incidence of Professional Rugby League Athletes. Int J Sports Physiol Perform. 2017;12(6):819-24.

339. Ullah S, Gabbett TJ, Finch CF. Statistical modelling for recurrent events: an application to sports injuries. Br J Sports Med. 2014;48(17):1287-93.

340. Windt J, Gabbett TJ, Ferris D, Khan KM. Training load--injury paradox: is greater preseason participation associated with lower in-season injury risk in elite rugby league players? Br J Sports Med. 2017;51(8):645-50.

341. Cummins C, Welch M, Inkster B, Cupples B, Weaving D, Jones B, et al. Modelling the relationships between volume, intensity and injury-risk in professional rugby league players. Journal of Science and Medicine in Sport. 2019;22(6):653-60.

342. Driller M, Cupples B. Sleep prior to and following competition in professional rugby league athletes. Science and Medicine in Football. 2019;3(1):57-62.

343. Caia J, Scott TJ, Halson SL, Kelly VG. Do players and staff sleep more during the pre- or competitive season of elite rugby league? Eur J Sport Sci. 2017;17(8):964-72.

344. Conlan G, McLean B, Kemp J, Duffield R. Effect of Training/Competition Load and Scheduling on Sleep Characteristics in Professional Rugby League Athletes. J Strength Cond Res. 2022;36(12):3390-7.

345. Thornton HR, Duthie GM, Pitchford NW, Delaney JA, Benton DT, Dascombe BJ. Effects of a 2-Week High-Intensity Training Camp on Sleep Activity of Professional Rugby League Athletes. Int J Sports Physiol Perform. 2017;12(7):928-33.

346. Thornton HR, Delaney JA, Duthie GM, Dascombe BJ. Effects of Preseason Training on the Sleep Characteristics of Professional Rugby League Players. Int J Sports Physiol Perform. 2018;13(2):176-82.

347. Caia J, Halson SL, Scott TJ, Kelly VG. Intra-individual variability in the sleep of senior and junior rugby league athletes during the competitive season. Chronobiol Int. 2017;34(9):1239-47.

348. Caia J, Thornton HR, Kelly VG, Scott TJ, Halson SL, Cupples B, et al. Does self-perceived sleep reflect sleep estimated via activity monitors in professional rugby league athletes? J Sports Sci. 2018;36(13):1492-6.

349. Dowell TL, Waters AM, Usher W, Farrell LJ, Donovan CL, Modecki KL, et al. Tackling Mental Health in Youth Sporting Programs: A Pilot Study of a Holistic Program. Child Psychiatry Hum Dev. 2021;52(1):15-29.

350. Nicholls AR, Madigan DJ, Fairs LRW, Bailey R. Mental health and psychological well-being among professional rugby league players from the UK. BMJ Open Sport Exerc Med. 2020;6(1):e000711.

351. Kola-Palmer S, Buckley S, Kingston G, Stephen J, Rodriguez A, Sherretts N, et al. “Someone to Talk to”: Influence of Player Welfare Provision on Mental Health in Professional Rugby League Players. Journal of Clinical Sport Psychology. 2019;13(3):486-503.

352. Kola-Palmer S, Lewis K, Rodriguez A, Kola-Palmer D. Help-Seeking for Mental Health Issues in Professional Rugby League Players. Front Psychol. 2020;11:570690.

353. Polman R, Nicholls AR, Cohen J, Borkoles E. The influence of game location and outcome on behaviour and mood states among professional rugby league players. Journal of Sports Sciences. 2007;25(13):1491-500.

354. Cupples B, O'Connor D, Cobley S. Facilitating transition into a high-performance environment: The effect of a stressor-coping intervention program on elite youth rugby league players. Psychology of Sport and Exercise. 2021;56:101973.

355. Golby J, Sheard M. Mental toughness and hardiness at different levels of rugby league. Personality and Individual Differences. 2004;37(5):933-42.

356. Green M, Morgan G, Manley A. Elite rugby league players’ attitudes towards sport psychology consulting. Sport &amp; Exercise Psychology Review. 2012.

357. Andrew M, O'Brien RW, Ford PR, Causer J. Developmental activities of professional male British rugby-league players versus controls. Sci Med Footb. 2022;6(3):381-8.

358. Cupples B, O’Connor D, Cobley S. Distinct trajectories of athlete development: A retrospective analysis of professional rugby league players. Journal of Sports Sciences. 2018;36(22):2558-66.

359. Cobley S, Hanratty M, O'Connor D, Cotton W. First Club Location and Relative Age as Influences on Being a Professional Australian Rugby League Player. International Journal of Sports Science & Coaching. 2014;9(2):335-46.

360. Hallinan CJ. Aborigines and Positional Segregation in Australian Rugby League. International Review for the Sociology of Sport. 1991;26(2):69-79.

361. Brown AD, Coupland C. Identity Threats, Identity Work and Elite Professionals. Organization Studies. 2015;36(10):1315-36.

362. Till K, Cobley S, Wattie N, O'Hara J, Cooke C, Chapman C. The prevalence, influential factors and mechanisms of relative age effects in UK Rugby League. Scand J Med Sci Sports. 2010;20(2):320-9.

363. Fleming S, Hardman A, Jones C, Sheridan H. ‘Role models’ among elite young male rugby league players in Britain. European Physical Education Review. 2005;11(1):51-70.

364. Rothwell M, Rumbold JL, Stone JA. Exploring British adolescent rugby league players’ experiences of professional academies and dropout. International Journal of Sport and Exercise Psychology. 2020;18(4):485-501.

365. Jones R, Mahoney J, Gucciardi F. On the Transition Into Elite Rugby League: Perceptions of Players and Coaching Staff. Sport, Exercise, and Performance Psychology. 2013;3:28.
